# The Inflamm-Aging Model Identifies Key Risk Factors in Atherosclerosis

**DOI:** 10.3389/fgene.2022.865827

**Published:** 2022-05-30

**Authors:** Yudan He, Yao Chen, Lilin Yao, Junyi Wang, Xianzheng Sha, Yin Wang

**Affiliations:** ^1^ Department of Biomedical Engineering, School of Intelligent Sciences, China Medical University, Shenyang, China; ^2^ Tumor Etiology and Screening Department of Cancer Institute and General Surgery, The First Affiliated Hospital of China Medical University, Shenyang, China

**Keywords:** inflamm-aging, atherosclerosis, immune homeostasis, causal analysis, sensitive analysis

## Abstract

**Background:** Atherosclerosis, one of the main threats to human life and health, is driven by abnormal inflammation (i.e., chronic inflammation or oxidative stress) during accelerated aging. Many studies have shown that inflamm-aging exerts a significant impact on the occurrence of atherosclerosis, particularly by inducing an immune homeostasis imbalance. However, the potential mechanism by which inflamm-aging induces atherosclerosis needs to be studied more thoroughly, and there is currently a lack of powerful prediction models.

**Methods:** First, an improved inflamm-aging prediction model was constructed by integrating aging, inflammation, and disease markers with the help of machine learning methods; then, inflamm-aging scores were calculated. In addition, the causal relationship between aging and disease was identified using Mendelian randomization. A series of risk factors were also identified by causal analysis, sensitivity analysis, and network analysis.

**Results:** Our results revealed an accelerated inflamm-aging pattern in atherosclerosis and suggested a causal relationship between inflamm-aging and atherosclerosis. Mechanisms involving inflammation, nutritional balance, vascular homeostasis, and oxidative stress were found to be driving factors of atherosclerosis in the context of inflamm-aging.

**Conclusion:** In summary, we developed a model integrating crucial risk factors in inflamm-aging and atherosclerosis. Our computation pipeline could be used to explore potential mechanisms of related diseases.

## Introduction

The burden of cardiovascular disease (CVD) has increased, leading to it being considered one of the most expensive diseases (i.e., seriously costing the economic burden) (Lopez et al., 2021). CVD is becoming an increasingly serious problem, with a high incidence rate and accounting for nearly one-third of total deaths (Stewart et al., 2017). Atherosclerosis is considered to be the major cause of CVD (Pahwa and Jialal, 2021). A chronic disease, atherosclerosis, involves the accumulation of plaques, and its major traditional risk factors (i.e., blood lipid proteasemia, diabetes, smoking, hypertension, and genetic abnormalities) have been reported by many epidemiological studies (Bergheanu et al., 2017; Pahwa and Jialal, 2021). However, atherosclerosis is a complex disease, and there are multiple cellular and molecular interactions involved in the progression of atherosclerosis that need to be further investigated and integrated (Lusis, 2000). Moreover, treatment is often difficult, expensive, invasive, and risky, and there is still no effective means of preventing atherosclerosis (Fogoros and Ali, 2020).

An increasing number of reports show that aging is a driving factor of atherosclerosis (El Assar et al., 2012; Wang and Bennett, 2012). Aging is closely related to endothelial dysfunction and arterial stiffness, which are considered to be early events leading to CVD. The aging process involves promotion of a series of risk factors (i.e., oxidative stress, endothelial dysfunction, and pro-inflammatory cytokines), leading to endothelial dysfunction and vascular system damage. In addition, cellular aging induces the release of microbubbles, further contributing to the development and calcification of atherosclerotic plaques (Alique et al., 2018). Therefore, the incidence of atherosclerosis increases with chronological age, and accelerated aging is the main risk factor for the development of atherosclerotic plaques (Kahn, 2021). Furthermore, atherosclerotic plaques represent a key index of cellular aging, which is characterized by reduced cell proliferation, increased DNA damage, and telomere shortening. In summary, a growing body of evidence indicates that atherosclerosis is promoted by cellular aging (Wang and Bennett, 2012).

Atherosclerosis is closely related to inflamm-aging, along with oxidative stress, endothelial dysfunction, and inflammation (Alique et al., 2018). Inflamm-aging is defined as a chronic inflammatory process during aging and mainly characterized by chronic progressive strengthening of the pro-inflammatory response (Xia et al., 2016). In other words, inflamm-aging promotes the body’s pro-inflammatory status with advancing aging, which is closely related to many aging diseases (Xia et al., 2016). A series of studies have shown that aging can promote atherosclerosis by damaging the connection between mitochondrial function and the intravascular inflammatory pathway (Tyrrell and Goldstein, 2020). For example, chronic inflammation is the main cause of age-related atherosclerosis, possibly exerting its effect through the IL-6 signaling pathway (Tyrrell and Goldstein, 2020). Furthermore, inflammatory factors released by senescent cells result in a senescence-associated secretory phenotype and even atherosclerosis (Childs et al., 2017). Selective targeting and elimination of these senescent cells (called heterolysis) has been shown to slow the growth of atherosclerotic lesions by reducing the release of inflammatory and adhesion factors (Childs et al., 2017).

**FIGURE 1:**
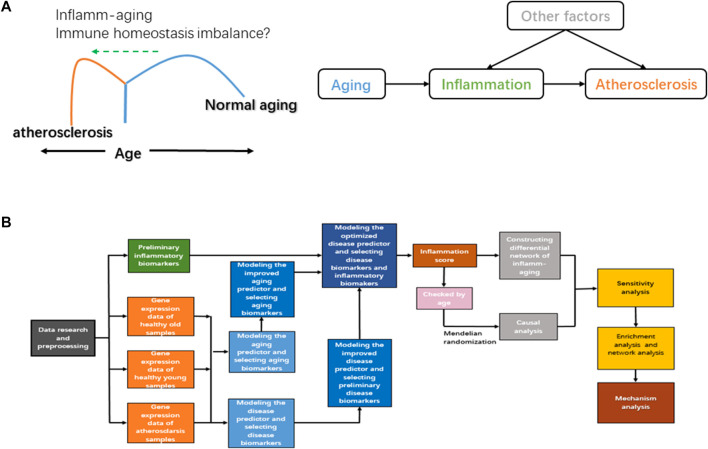
**(A)** Diagram of the hypothetical mechanism of atherosclerosis. **(B)** Workflow of our study.

Inflamm-aging induces an immune homeostasis imbalance during atherosclerosis development. As a chronic inflammatory disease, atherosclerosis is characterized by endothelial dysfunction and abnormal immune responses; immune cells and lipids are also involved (Karunakaran et al., 2015). The normal function of the adaptive immune system appears to decline with chronological age, and an advanced inflammatory response is promoted, leading to the imbalance of immune homeostasis (Sadighi Akha, 2018). Both innate and adaptive immune responses have been identified in atherosclerosis, and components of cholesterol-carrying low-density lipoprotein can further trigger abnormal inflammation (Keesey and Powley, 2008). Unsaturated fatty acids can also regulate both the differentiation and activation of T cells and then induce inflammation and oxidative stress (Hubler and Kennedy, 2016), and accumulation of dysregulated proteins can damage the normal function of the endoplasmic reticulum, which has an important role in oxidative stress and the immune response (So, 2018). In short, the nutritional balance is critical to immune homeostasis (Chandra, 1997). Chronic oxidative stress is also vital to immune/inflammatory cells during the inflamm-aging process (Fuente and Miquel, 2009). However, further and more systematic investigation is required to fully elucidate how atherosclerosis is triggered by inflamm-aging in the context of immune homeostasis imbalance. In order to study the potential mechanisms involved in inflamm-aging, immune homeostasis imbalance, and atherosclerosis, we hypothesized that the progression of atherosclerosis would be promoted by accelerated chronic inflammation during the aging process when immune homeostasis was disrupted (Figure 1A).

At present, there are many omics profiles to be further analyzed; thus, proper prediction models need to be developed (Munger et al., 2021). Machine learning (ML), which is regarded as an extension of classical statistical modeling, can digest large amounts of data to identify high-order correlations and generate predictions (Alber et al., 2019; Zhavoronkov et al., 2019). For example, the supervised ML method can be used to understand the aging process (Fabris et al., 2017). Recently, an ML-derived score has been reported to show satisfactory performance in the prediction of cardiovascular events (Sánchez-Cabo et al., 2020). Some studies have also shown that ML can predict CVD and identify key markers related to inflamm-aging and CVD (Alalawi and Alsuwat, 2021; Munger et al., 2021; Sayed et al., 2021). Network analysis can also be used to find clusters of highly related genes and to associate modules with each other and with external sample traits (Deshpande et al., 2016). Recently, causality analysis was applied to CVD and found to be informative, using Mendelian randomization (MR) to integrate genomic and phenotypic information (Yazdani et al., 2016). Although many studies have identified CVD-related risk markers (Shadrina et al., 2020), the inflamm-aging mechanism in atherosclerosis (thus the casual relationship between inflamm-aging and atherosclerosis as well as relative potential risk factors) needs to be explored more thoroughly. Therefore, there is an urgent need to model atherosclerosis progression based on the inflamm-aging process, integrating the potential mechanisms related to immune homeostasis imbalance at a system level.

In order to further explore the inflamm-aging mechanism in atherosclerosis, we present a computational pipeline using ML and systems biology methods (Figure 1B), comprising the following steps (Lopez et al., 2021): we developed an improved model of inflamm-aging-related predictors (the inflamm-aging model) of atherosclerosis and identified relative risk markers (Stewart et al., 2017). An inflamm-aging score was calculated by integrating both aging and inflammatory markers (Pahwa and Jialal, 2021). MR was carried out to explore the causal relationship between inflamm-aging and atherosclerosis (Bergheanu et al., 2017). An inflamm-aging differential network was constructed by summarizing the (partial) correlation between each pair of genes based on the inflamm-aging score (Lusis, 2000). The Markov chain Monte Carlo (MCMC) method was used to further explore a sensitivity index for inflamm-aging, immune homeostasis imbalance, and atherosclerosis (Fogoros and Ali, 2020). Enrichment analysis and network analysis were carried out to study potential risk factors in atherosclerosis. As a result, the casual relationship between inflamm-aging and atherosclerosis was identified, and potential mechanisms related to nutritional balance, oxidative stress, and vessel homeostasis were integrated based on our computational pipeline.

## Results

### Modeling Inflamm-Aging Predictors and Identifying Relative Risk Markers

**FIGURE 2:**
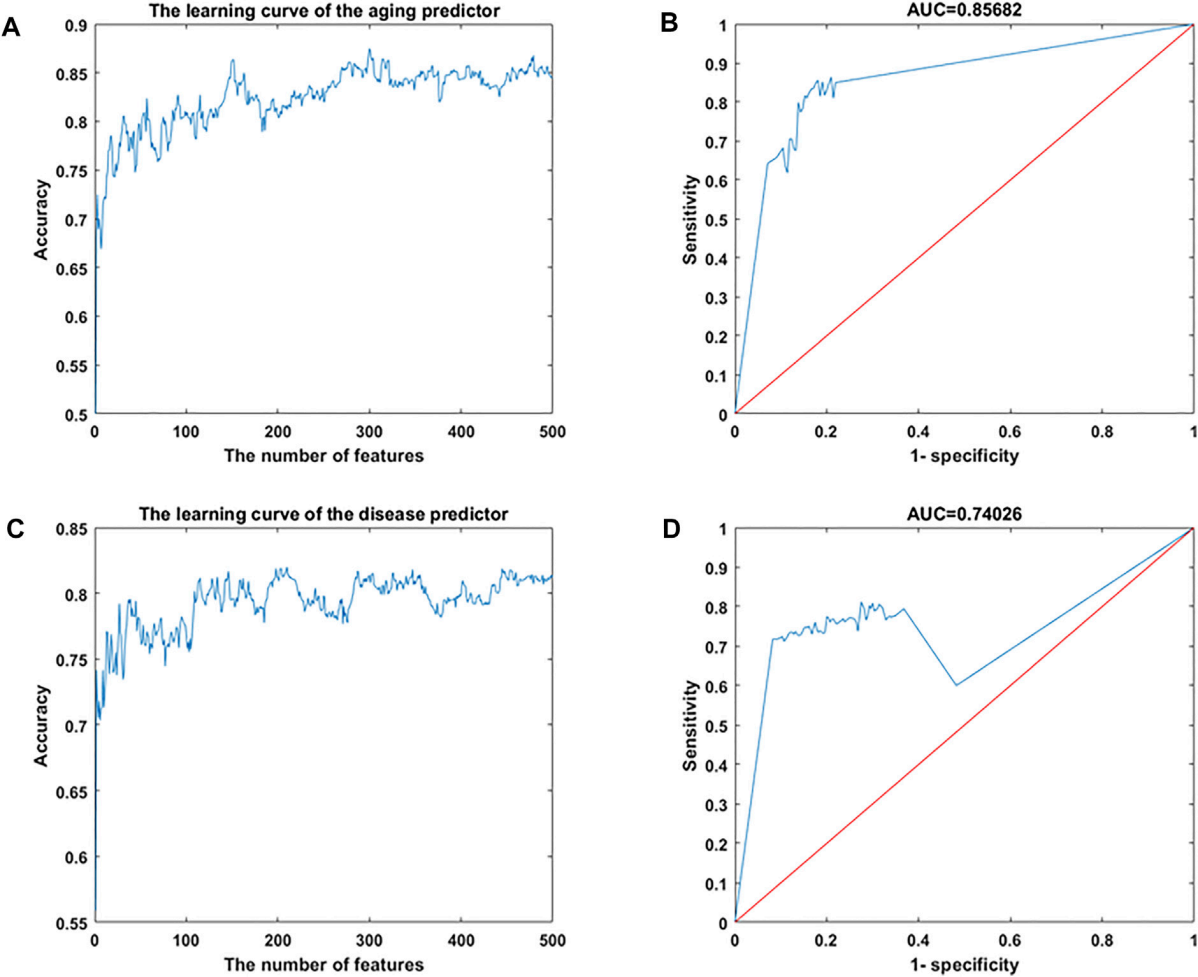
Machine learning results. **(A,B)** Aging predictor from our previous study, selecting the number of aging markers. **(C,D)** Improved inflamm-aging predictor, selecting the number of disease markers. **(A,C)** Learning curve for the training dataset. **(B,D)** ROC curve for the test dataset.

Gene expression data were downloaded from the Gene Expression Omnibus (GEO) database, including 926 samples and 11,313 genes (Supplementary Tables S1, S2). The RelifF algorithm was used to sort relative genes, and the k-nearest neighbors (kNN; *k* = 5 with the cosine distance) algorithm was selected to mode aging predictors and disease predictors (determined by 10-fold cross-validation; results in Supplementary Tables S3–S7). Other classification results are shown in Supplementary Tables S8–S10 based on the support vector machine (SVM), naive Bayes (NB), and ensemble learning (integrating 100 decision tree models) algorithms (Supplementary Tables S8–S10). The accuracy of the models was then validated using test data (Figure 2; Supplementary Figure S1; Table 1) including 126 atherosclerosis samples and 170 controls, where the aging/inflammatory/disease as well as relative parameters (selected by the cross-validation based on the training set, in Supplementary Tables S5) were also used in the test set. Furthermore, top-ranked aging, inflammatory, and disease markers were used in the improved inflamm-aging predictor (Supplementary Tables S5). The aging predictor accuracy was 0.7017, and 441 aging markers were identified; the prediction accuracy of the improved inflamm-aging model was 0.7037, with 186 inflammatory markers and 316 disease markers identified (see Materials and Methods; relative parameters in Supplementary Tables S5). The area under the receiver operating characteristic (ROC) curve values were 0.85682 and 0.74206 for the aging predictor and the improved inflamm-aging predictor, respectively. These results showed that our predictors were reliable and able to identify healthy young and old samples as well as distinguish between normal and disease samples with sufficient accuracy (Figure 2; Table 1).

**TABLE 1:** Accuracy of the predictor on the training and test datasets.

	Accuracy on the training dataset	Accuracy on the test dataset
Traditional aging predictor	0.7881	0.6278
Aging predictor from our previous study	0.8310	0.7017
Traditional disease predictor	0.8096	0.6699
Disease predictor from our previous study	0.8018	0.6801
Improved inflamm-aging predictor	0.7989	0.7037

Various biomarkers of aging/disease have been associated with biological functions (Supplementary Table S5). For example, MMP8, the top aging marker, is a collagenase with proteolytic activity against matrix proteins and is an important participant in the function of the vascular system and the development of atherosclerosis (Laxton et al., 2009; Toba et al., 2017; Yu et al., 2019). Its expression in the heart increases with chronological age, enabling it to regulate signal transduction during the aging process by regulating the expression and activity of cytokines, chemokines, growth factors, hormones, and angiogenic factors (Mota et al., 2018). The top disease marker is KIR3DL1. Killer immunoglobulin-like receptors (KIR) encode receptors expressed on some subsets of natural killer cells and T lymphocytes (Niepiekło-Miniewska et al., 2014), which can regulate inflammatory response (Ross, 2014; Pugh et al., 2019). Among the 186 inflammatory markers identified, HSPA6 had the lowest false discovery rate (FDR = 4.38e-9). HSPA6 participates in cell protection (Noonan et al., 2007) and cell proliferation and affects the receptor binding affinity of IgG (Takai, 2002; Wang et al., 2020a). HSPA6 is also a putative target of endothelial nitric oxide synthase and is involved in the highly clinically relevant inhibition of vascular smooth muscle cell (VSMC) proliferation, which can repair vessels in human atherosclerosis (McCullagh et al., 2016). Thus, it could be speculated that VSMCs in the vascular system are closely related to atherosclerosis development in the context of inflamm-aging, consistent with previous research results (Bennett et al., 2016).

### Comparison of Inflamm-Aging Scores Between Normal and Disease Samples

**FIGURE 3:**
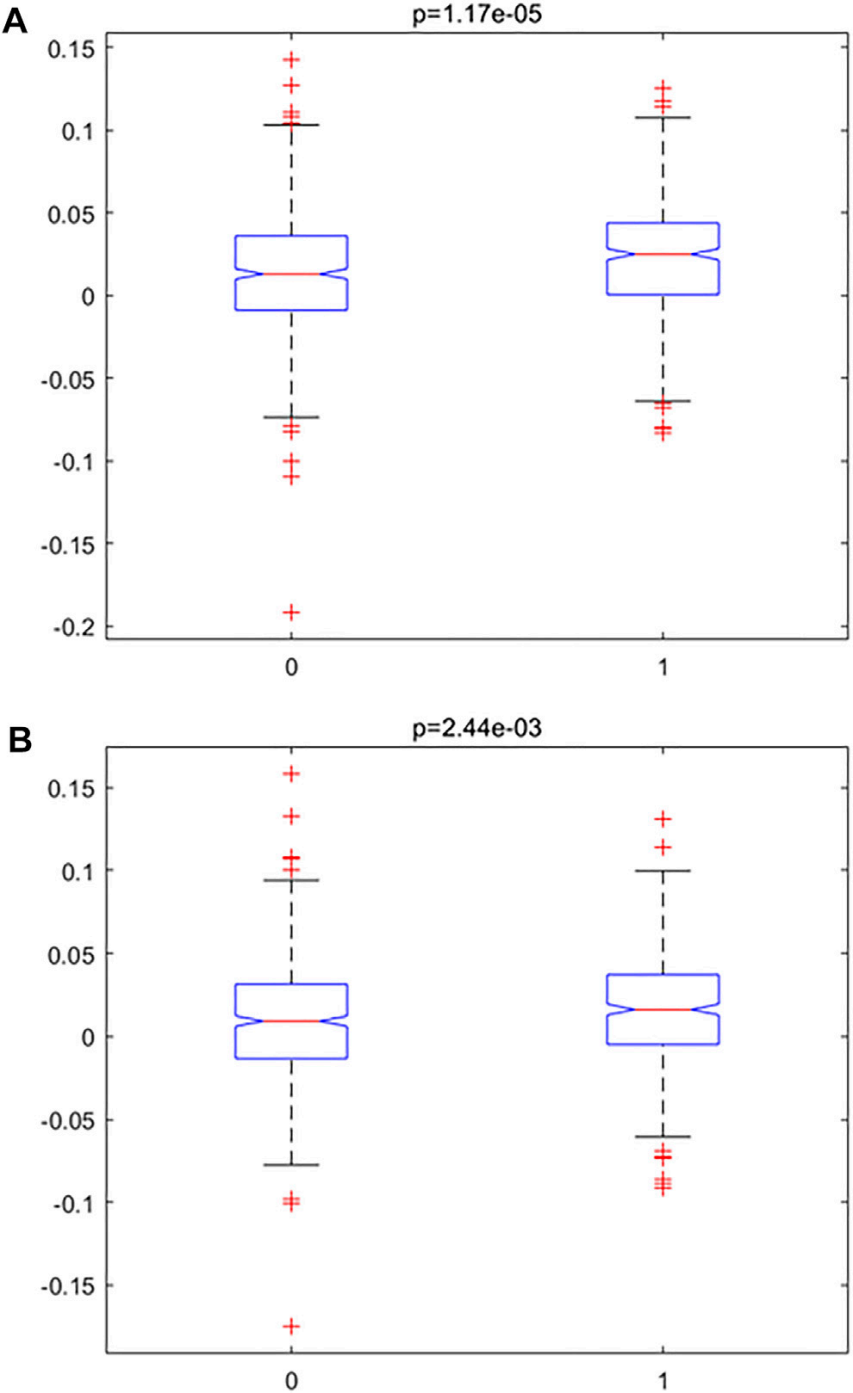
Accelerated inflamm-aging pattern using the Kruskal–Wallis test. **(A)** Original inflamm-aging score. **(B)** Adjusted inflamm-aging score.

In order to investigate the accelerated inflamm-aging pattern in atherosclerosis, an inflamm-aging score was constructed by integrating 186 inflammatory markers and 441 aging markers based on the training data (details are given in the “Materials and Methods” section); the results are shown in Table 2. Both the median and mean of the inflamm-aging scores of atherosclerosis samples were greater than those of control samples. These results indicated an accelerated inflamm-aging pattern in atherosclerosis, consistent with previous findings (Lavin Plaza et al., 2020). To further verify this conclusion, the Kruskal–Wallis test (Figure 3A) was used to validate the significance of inflamm-aging in atherosclerosis. The results confirmed that atherosclerosis samples showed a significantly accelerated inflamm-aging pattern compared with normal samples (*p* = 1.6856e-05). In addition, when inflamm-aging scores were adjusted by chronological age (see Materials and Methods), the accelerated inflamm-aging pattern remained significant (*p* = 0.0024; Figure 3B; Table 2). A principal component analysis (PCA) plot is also shown in Supplementary Figure S2.

**TABLE 2:** Inflamm-aging scores of the disease and control groups.

	Mean (control)	Mean (disease)	Median (control)	Median (disease)
Original score	0.0129	0.0230	0.0131	0.0250
Score adjusted by age	0.0096	0.0161	0.0092	0.0162

### Exploring the Causal Relationship Between Inflamm-Aging and Atherosclerosis by MR

In order to study the inflamm-aging mechanisms in atherosclerosis more thoroughly, the MR method was used to distinguish causal relationships among aging, inflammation, and atherosclerosis, and partial correlation coefficients were calculated (see Materials and Methods for details). Crucial causal relationships related to inflamm-aging were identified in this way (Table 3, Supplementary Table S1, S2). For example, RPRM was the top aging marker in MR, related to the most (Takai, 2002) inflamm-aging markers. RPRM is a highly glycosylated cytoplasmic protein encoded by a p53-dependent putative tumor suppressor gene (Ohki et al., 2000). Its overexpression decreases cell proliferation, migration, and invasion and increases apoptosis (Figueroa et al., 2017). PECAM1 was the top disease marker, related to the maximum number (Merl-Pham et al., 2019) of aging markers. The expression of PECAM1 is associated with the response of various immune cell types (i.e., CD4 + T cells, B cells, CD8 + T cells, neutrophils, macrophages, and dendritic cells) as well as endothelial cells (Harry et al., 2008; Ye et al., 2021). The activation of endothelial PECAM1 has been shown to lead to nuclear translocation of nuclear factor κB (NF-κB) and expression of inflammatory and adhesion mediators, which promoted atherosclerosis (Harry et al., 2008). Polymorphism of PECAM1 is associated with the incidence rate of coronary atherosclerosis (Harry et al., 2008), indicating that inflamm-aging could accelerate atherosclerosis progression.

**TABLE 3:** Top 10 pairs with the largest absolute difference in correlation and partial correlation between “inflammation+ atherosclerosis−” and “inflammation+ atherosclerosis+”.

Aging marker	Disease marker	Difference
BIRC2	PLOD1	0.0650
CRBN	IRF5	0.0617
ICAM2	IRF5	0.0581
BIRC2	CPNE1	0.0524
RPRM	CEBPA	0.0518
ICAM2	NGB	0.0514
ICAM2	CEBPA	0.0499
ICAM2	PARP16	0.0496
ICAM2	PECAM1	0.0491
ATP6V0C	POM121	0.0483

BIRC2–PLOD1 was the top aging–atherosclerosis pair (with the maximum differential K-S value of 0.06498; Table 3). BIRC2 is an E3 ubiquitin-protein ligase that regulates NF-κB signaling to maintain endothelial cell survival and blood vessel homeostasis during vascular development (Santoro et al., 2007; Samanta et al., 2020). PLOD1 can promote cell growth and the aerobic glycolysis process (Zhang et al., 2021). It also plays vital roles in atherosclerosis, including enhancing collagen fibril deposition (Yuan et al., 2021), modifying collagen (Wågsäter et al., 2013), and regulating the expression of extracellular matrix (ECM) (Merl-Pham et al., 2019). Thus, we conjectured that the vascular system would have an effect on atherosclerosis. In summary, key causal relationships between inflamm-aging and atherosclerosis were identified using MR.

### The Inflamm-Aging Differential Network Revealed Key Inflamm-Aging Mechanisms in Atherosclerosis

In order to explore potential inflamm-aging mechanisms in atherosclerosis at a system level, an inflamm-aging differential network was constructed by calculating the (partial) correlation coefficient for each pair of genes in the training data and test data, respectively (details shown in Materials and Methods). The Pearson correlation coefficient of degree and degree frequency after logarithmic transformation was -0.8827 (*p* = 2.7871e-30; Supplementary Figure S3), with a small ratio of genes having a large degree. That is, the network had the scale-free characteristic. Furthermore, when the network was verified on the test set, the *p*-value was close to 0 using Fisher’s exact test. All these results indicate the reliability of the network.

The network markers indicated that endothelium, oxidative stress, and inflammation have important roles in atherosclerosis; the top-ranked genes (by degree) and their corresponding functions are shown in Table 4 (Yang et al., 2000; Zhang, 2013; Schisler et al., 2015; Mezzadra et al., 2017; Zheng et al., 2017; Guan et al., 2018; Lan et al., 2020; Nie et al., 2020; Zheng et al., 2020; Chen et al., 2021a; Cao et al., 2021; Covarrubias et al., 2021; Liu et al., 2021; Wang et al., 2021; Zheng et al., 2021). For example, the gene with the largest degree was PXN, which encodes a cytoskeleton protein. PXN is related to the attachment of actin membrane to ECM, tissue remodeling, and cell proliferation and survival. PXN also has important roles in focal adhesion, endothelial dysfunction, inflammation, and oxidative stress (Chen et al., 2021a).

**TABLE 4:** Top 10 markers with the highest degrees.

Gene symbol	Degree	Function	Reference
PXN	117	1) Related to the attachment of actin membrane to ECM	Chen et al. (2021a)
		2) Participates in tissue remodeling, cell proliferation, and survival	
		3) Important role in focal adhesion, endothelial dysfunction, inflammation, and oxidative stress	
ZNF22	110	Induces cell apoptosis	Schisler et al. (2015), Nie et al. (2020)
RBM10	100	1) Regulates cell apoptosis, cell proliferation, cell invasion and metastasis, and inflammatory response	Cao et al. (2021)
		2) Affects the pathogenesis of atherosclerosis	
NADK	93	1) Important role in regulating cell aging and aging-related diseases	Zhang (2013), Covarrubias et al. (2021)
		2) Responsible for the production of NADP in the cytoplasmic matrix and participates in the counteraction of oxidative damage	
ANKRD12	90	Participates in bone marrow mesenchymal stem cell differentiation, including cardiac muscle cells, nerve cells, blood cells, and myogenic cells	Wang et al. (2021)
PJA2	89	Promotes M1 macrophage polarization, M2 to M1 macrophage transformation, and the inflammatory response	Zheng et al. (2021)
ATP5I	89	Induces oxidative stress and DNA damage	Liu et al. (2021)
MAP3K3	88	1) Necessary for angiogenesis	Yang et al. (2000), Zheng et al. (2017)
		2) Related to endothelial cell proliferation and apoptosis and interacts with heart and myocardium	
		3) Participates in cell proliferation, differentiation, and apoptosis	
		4) Related to cellular aging	
CMTM6	84	1) Important role in regulating immune response and inflammatory activation	Mezzadra et al. (2017), Guan et al. (2018), Zheng et al. (2020)
		2) Regulates the expression of PD-L1. PD-L1 can inhibit the function of T-cell inhibition	
		3) Promotes cell migration and invasion	
SNRPG	82	1) Arrests the cell cycle	Lan et al. (2020)
		2) Activates p53 signaling	

### Sensitivity Analysis Further Highlighted the Immune Homeostasis Imbalance in Atherosclerosis

In order to further investigate the mechanisms underlying inflamm-aging, immune homeostasis imbalances, and atherosclerosis, the MCMC method was used to develop a sensitivity index for the relationship between inflamm-aging and atherosclerosis (based on the results obtained by MR). A bipartite graph of aging–disease pairs was constructed (see Materials and Methods, and Supplementary Figure S4).

The top 10 most sensitive pairs are shown in Table 5 (based on absolute differential frequencies). The relationship pair with the greatest difference (0.08172) was CRBN–MRPL40 (Table 5). CRBN functions as a potential regulator of cell homeostasis by mediating the ubiquitination of target substrates involved in processes including ion transport, the AMP-activated protein kinase (AMPK) signaling pathway, and cellular metabolisms (Shi and Chen, 2017; Jeon et al., 2021). It also protects cells from fat metabolism disorders (induced by high-fat levels) and negatively regulates CD4^+^ T cell activation (Shi and Chen, 2017), and has been implicated in a variety of diseases including CVD, obesity, and fatty liver (Shi and Chen, 2017). MRPL40 is related to mitochondrial functions (Li et al., 2019) and is crucial to ensuring ribosome translational fidelity and subsequent assembly of the oxidative phosphorylation complex (Jia et al., 2009). In short, these results indicate that oxidative stress plays an important part in the development of atherosclerosis.

**TABLE 5:** Top 10 pairs with the largest absolute difference between the frequency of disease inflammation and the frequency of the healthy inflammation in the relationship pairs with the shortest paths.

Aging marker	Disease marker	Difference
CRBN	MRPL40	0.0817
RDX	INPP5E	0.0774
ENOPH1	IRF5	0.0744
TACC2	CEBPA	0.0720
CKAP4	CEBPA	0.0678
MMP11	ARPC2	0.0678
MCL1	RBBP5	0.0645
HERC6	UBE2G2	0.0645
PURA	UBE2G2	0.0635
ATP6V0C	POM121	0.0614

The aging marker related to the most (Keesey and Powley, 2008) disease markers (Supplementary Tables S13) was HEY2, the main sensor of Notch signal transduction. HEY2 has a key role in cardiac development; for example, it protects the heart from age-induced hypertrophic changes (Röning et al., 2022). It also regulates the proliferation of VASCs (Shirvani et al., 2007). PECAM1 was the inflamm-aging marker related to the maximum number (Alber et al., 2019) of aging markers (Supplementary Tables S14) in both the sensitivity analysis and MR (Harry et al., 2008; Ye et al., 2021). These results highlighted the critical role of the immune homeostasis imbalance in atherosclerosis progression.

### Underlying Inflamm-Aging Mechanisms Based on Enrichment Analysis

In order to further explore the potential mechanism linking inflamm-aging and atherosclerosis, the shortest path of each aging–disease pair (as identified by MR or MCMC) in the inflamm-aging differential network was determined using the Dijkstra algorithm; then, the Kyoto Encyclopedia of Genes and Genomes (KEGG) pathway analysis and gene ontology (GO) enrichment analysis with respect to biological process (BP) was performed on these shortest paths.

**FIGURE 4:**
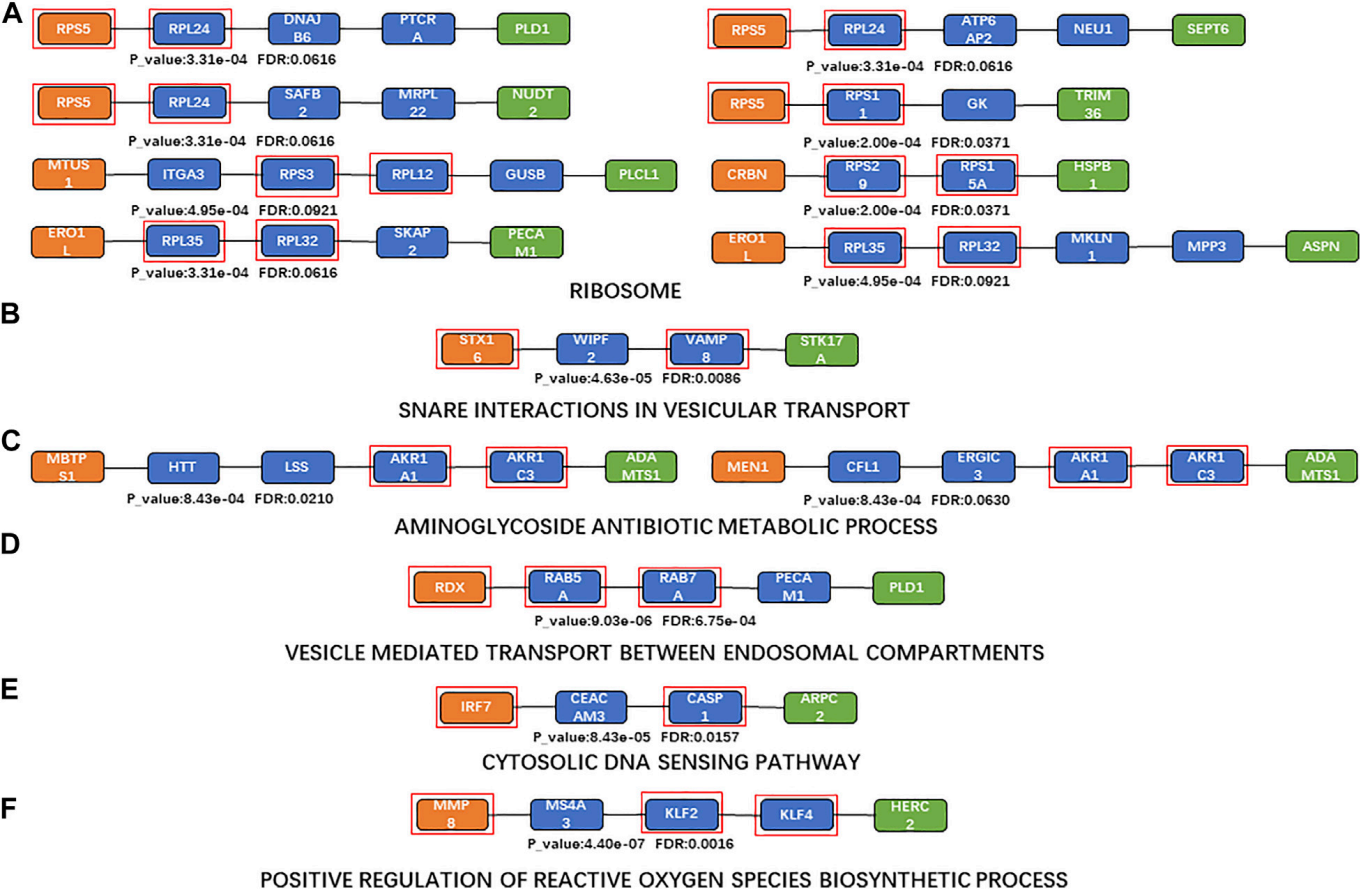
Shortest paths for enrichment analysis. **(A)**a Maximum number of enriched paths for the KEGG pathway (“ribosome”); **(B)**KEGG pathway with the minimum FDR (“SNARE interactions in vesicular transport”); **(C)** maximum number of enriched paths for BP terms (“aminoglycoside antibiotic metabolic process” (GO:0030647)); **(D)** BP term with the minimum FDR (“vesicle-mediated transport between endosomal compartments” (GO:009,892)); **(E)** KEGG pathway with the minimum FDR based on sensitivity analysis (“cytosolic DNA-sensing pathway”); **(F)** BP term with the minimum FDR based on sensitivity analysis (“positive regulation of reactive oxygen species biosynthetic process” (GO:1903428)). Orange nodes, aging biomarkers; blue nodes, genes connecting aging biomarkers and atherosclerosis biomarkers; green nodes, atherosclerosis biomarkers; genes in the red square frames, genes with enriched functions.

The top 10 KEGG pathways (those enriched in the shortest paths) are shown in Table 6 (Turi et al., 2019; Jia et al., 2021; Chothani et al., 2019; Yin et al., 2021; Manhas and Rath, 2020; Li et al., 2018; Nakajima et al., 2019; Hosseini et al., 2021; Critchley et al., 2018; Lathe et al., 2014; Margiotta, 2021; Nickel et al., 2002; Chen et al., 2020; Luo et al., 2020; Miller et al., 2021; Stadtman et al., 2005; Bonifácio et al., 2021; Blachier et al., 2020; Kumar et al., 2017; Récher, 2021; Klemm and Ruland, 2006). The most frequently enriched KEGG pathway was “ribosome” (enriched in eight shortest paths; Figure 4A). The rate of ribosome biogenesis has an important role in cell cycle progression and proliferation, which are closely related to coronary restenosis and atherosclerosis (Turi et al., 2019; Jia et al., 2021). Further, RNA translation and RNA transcription in the ribosome participate in fibration, which is vital to the development of atherosclerosis (Chothani et al., 2019). Advanced inflamm-aging impairs ribosome biogenesis, leading to autoantibody production against ribosomal proteins (Yin et al., 2021). Increased oxidative stress may also damage ribosomal RNA (Manhas and Rath, 2020). In short, the ribosome has an important role in the development of atherosclerosis.

**TABLE 6:** Top 10 KEGG with the most numerous enriched paths.

KEGG	Enriched shortest path	Score	Function	Reference
Ribosome	8	7.4949	1) Important role in cell cycle progression and proliferation, closely related to coronary restenosis and atherosclerosis	Chothani et al. (2019), Turi et al. (2019), Manhas and Rath (2020), Jia et al. (2021), Yin et al. (2021)
			2) Regulates fibrosis, which is an important factor in the occurrence of atherosclerosis	
			3) With increasing age, abnormality of the immune system will affect the biogenesis of ribosomes and lead to the production of ribosomal protein autoantibodies	
			4) Increases oxidative stress during aging, eventually causing damage to ribosomal RNA	
Parkinson’s disease	7	6.5642	Cardiovascular disease and Parkinson’s disease share common risk factors	Li et al. (2018)
Oxidative phosphorylation	5	4.7213	1) Regulates adiponectin secretion in epicardial adipose tissue and has anti-atherosclerotic and anti-inflammatory effects on blood vessels	Nakajima et al. (2019), Hosseini et al. (2021)
			2) Triggers atherosclerosis by affecting mitochondrial functions	
Huntington’s disease	4	3.7964	Myocardial dysfunction and vasoconstrictor dysfunction are involved in Huntington’s disease progression	Critchley et al. (2018)
Alzheimer’s disease	3	2.8729	Both atherosclerosis and Alzheimer’s disease are involved in inflammation, macrophage infiltration, and vascular system obstruction	Lathe et al. (2014)
SNARE interactions in vesicular transport	1	0.9914	1) SNARE protein is an essential component that allows membrane fusion and can regulate vesicle fusion	Nickel et al. (2002), Chen et al. (2020), Margiotta (2021)
			2) SNARE has a key role in cell homeostasis. Vesicle transport is the main mechanism of protein and lipid exchange between membrane-bound organelles in eukaryotic cells	
			3) Vesicle transport in mitochondria affects mitochondrial function, which is one of the characteristics of aging	
Cytosolic DNA-sensing pathway	1	0.9843	1) Promotes the expression of immune genes	Luo et al. (2020), Miller et al. (2021)
			2) Cytoplasmic DNA and cytoplasmic DNA-sensing adapter STING play a key part in aortic degeneration by promoting smooth muscle cell damage, macrophage MMP production, and ECM degeneration	
			3) Endogenous cytoplasmic DNA is a contributing factor to inflammation in the absence of pathogenic infection, which is associated with many chronic age-related diseases, including cancer, cardiovascular disease, and neurodegenerative diseases	
Cysteine and methionine metabolism	1	0.9785	1) The accumulation of oxidized methionine residues of protein is related to aging	Stadtman et al. (2005), Kumar et al. (2017), Blachier et al. (2020), Bonifácio et al. (2021)
			2) Hyperhomocysteinemia and increased circulating levels of homocysteine (Hcy) are generally considered to be independent risk factors for peripheral atherosclerosis	
			3) Hcy can cause oxidative stress, inflammation, and endothelial dysfunction	
			4) Cysteine is an important source of energy and biomass and has a central role in cell metabolism	
			5) The metabolites of cysteine and methionine are related to fat metabolism and cardiovascular disease	
Acute myeloid leukemia	1	0.9752	Inflammation can affect the progression of myeloid leukemia	Récher (2021)
FC epsilon RI signaling pathway	1	0.9692	Can induce the production of pro-inflammatory factors and mast cell degranulation	Klemm and Ruland (2006)

The top enriched KEGG pathway (with the minimum FDR) was “SNARE interactions in vesicular transport” (*p* = 4.6346 and FDR = 0.0086; Figure 4B). The SNARE protein is an essential component in the regulation of vesicle fusion and plays a key part in maintaining cell homeostasis (Margiotta, 2021). Vesicle transport is the main mechanism of protein and lipid exchange between membrane-bound organelles in eukaryotic cells (Nickel et al., 2002); vesicle transport in mitochondria also affects mitochondrial function, which is one of the key characteristics of aging (Chen et al., 2020). Extracellular vesicles are involved in various processes including inflammation, coagulation, vascular dysfunction, angiogenesis, and aging, which contribute to the occurrence and progression of atherosclerotic thrombotic diseases (Konkoth et al., 2021).

The top 10 BP terms (those enriched in the most paths) are shown in Table 7 (Krause et al., 2016; Sainlos et al., 2003; Gonzalez and Spencer, 1998; Swirski and Nahrendorf, 2014; Meng et al., 2022; De Stefano et al., 2021; Kurek et al., 2021; Kiechl et al., 1998; Tokarev et al., 2009; Hu et al., 2015; Van-Assche et al., 2011; Shimizu et al., 1997; Moore et al., 2013; Patel et al., 2000). “Aminoglycoside antibiotic metabolic process” (GO:0030647) was the most enriched BP term (enriched in two shortest paths; Figure 4C). Aminoglycosides provide a favorable scaffold for the synthesis of various cationic lipids (Krause et al., 2016; Sainlos et al., 2003), and are used to treat and prevent endocarditis (Gonzalez and Spencer, 1998). “Vesicle-mediated transport between endosomal compartments” (GO:0098927) was the BP term with minimum FDR (*p* = 9.0282e-08, FDR = 6.7540e-04; Figure 4D). The main communication process between a cell and its environment is vesicle transport, through bidirectional trafficking among the Golgi, endosomes, and lysosomes (Tokarev et al., 2009). The endosomal–lysosomal system and endomembrane system are also closely related to both lipid and protein metabolism (Hu et al., 2015).

**TABLE 7:** Top 10 BP with the most numerous enriched paths.

BP	Enriched shortest path	Score	Function	Reference
Aminoglycoside antibiotic metabolic process 0030647	2	1.9160	1) Aminoglycoside antibiotic works by inhibiting protein synthesis	Gonzalez and Spencer (1998), Sainlos et al. (2003), Krause et al. (2016)
			2) Aminoglycosides provide a favorable scaffold for the synthesis of various cationic lipids	
			3) Aminoglycosides are used to treat and prevent endocarditis	
Myeloid leukocyte activation GO:0002274	2	1.8743	1) Leukocytes can engender protective immunity and protect the host from damage	Swirski and Nahrendorf (2014)
			2) Important role in inflammation, immunity, and atherosclerosis	
Glycoside metabolic process GO:0016137	2	1.8682	1) Glycosides are associated with inflammation, oxidative stress, lipid metabolism, and atherosclerosis	De Stefano et al. (2021), Kurek et al. (2021), Meng et al. (2022)
			2) Flavonoid glycosides can protect vascular endothelial cells by inhibiting inflammation to restrict atherosclerosis	
			3) Luteolin-7-o-glucoside can reduce the activity of oxidative stress and inflammatory mechanisms in different physiological systems	
Tertiary alcohol metabolic process GO:1902644	2	1.8638	Alcohol affects the progress of atherosclerosis	Kiechl et al. (1998)
Vesicle-mediated transport between endosomal compartments GO:0098927	1	0.9993	1) The main communication process between the cell and its environment	Tokarev et al. (2009), Hu et al. (2015)
			2) Bi-directional trafficking between the Golgi, endosomes, and lysosomes connects the two major intracellular trafficking pathways	
			3) Endosomes gradually mature into lysosomes, and the acidification of late endosomes is accompanied by vesicle transport	
			4) Endosomal–lysosomal system and endomembrane system are related to lipid homeostasis and protein homeostasis	
Positive regulation of reactive oxygen species biosynthetic process GO:1903428	1	0.9984	1) Induces LDL oxidation and foam cell formation and activates many redox-sensitive transcription factors including NF-κB and AP1	Van-Assche et al. (2011)
			2) Regulates the expression of multiple promoters/anti-inflammatory genes involved in atherosclerosis	
Positive regulation of nitric oxide metabolic process GO:1904407	1	0.9984	Nitric oxide (NO) is associated with atherosclerosis as it inhibits the proliferation of VSMCs, monocyte/macrophage adhesion, platelet aggregation and adhesion, and LDL oxidation	Shimizu et al. (1997)
Regulation of nitric oxide metabolic process GO:0080164	1	0.9982	NO is associated with atherosclerosis as it inhibits the proliferation VSMCs, monocyte/macrophage adhesion, platelet aggregation and adhesion, and LDL oxidation	Shimizu et al. (1997)
Regulation of macrophage activation GO:0043030	1	0.9970	1) The number and phenotype of macrophages can affect the inflammatory state of plaques	Moore et al. (2013)
			2) Lipoprotein uptake by macrophages is considered to be one of the earliest pathogenic events in new plaques	
			3) The M2 polarization pathway of macrophages can prevent atherosclerosis	
Reactive nitrogen species metabolic process GO:2001057	1	0.9969	1) Affects endothelial function	Patel et al. (2000)
			2) Low-level proteins and lipids modified by reactive nitrogen can promote the development of atherosclerosis through mechanisms involving signal transduction	

Enrichment analysis was also performed based on the results of the sensitivity analysis. “Cytosolic DNA-sensing pathway” was the KEGG pathway with the minimum FDR in the sensitivity analysis (*p* = 8.4266e-05, FDR = 0.0157; Figure 4E); this can promote the expression of immune markers (Luo et al., 2020). Cytoplasmic DNA and cytoplasmic DNA-sensing adapter STING play key parts in aortic degeneration by promoting smooth muscle cell damage, matrix metalloproteinase (MMP) production in macrophages, and ECM degeneration (Luo et al., 2020). Endogenous cytoplasmic DNA is also a contributing factor to inflammation in the absence of pathogenic infection and is associated with many chronic age-related diseases, including CVD and neurodegenerative diseases (Miller et al., 2021). “Positive regulation of reactive oxygen species biosynthetic process” was the BP term with minimum FDR (GO:1903428, *p* = 4.3964e-07, FDR = 0.0016; Figure 4F). Reactive oxygen species (ROS) play a critical part in the regulation of the expression of multiple promoters and anti-inflammatory genes involved in atherosclerosis, *via* inducing low-density lipoprotein (LDL) oxidation, foam cell formation, and activation of many redox-sensitive transcription factors including NF-κB and activating protein 1 (Van-Assche et al., 2011).

In summary, a series of risk factors in atherosclerosis were identified, including inflammation, nutritional metabolism, energy metabolism, and the vascular system.

### Network Markers Revealed Crucial Mechanisms Between Aging and Atherosclerosis

**FIGURE 5:**
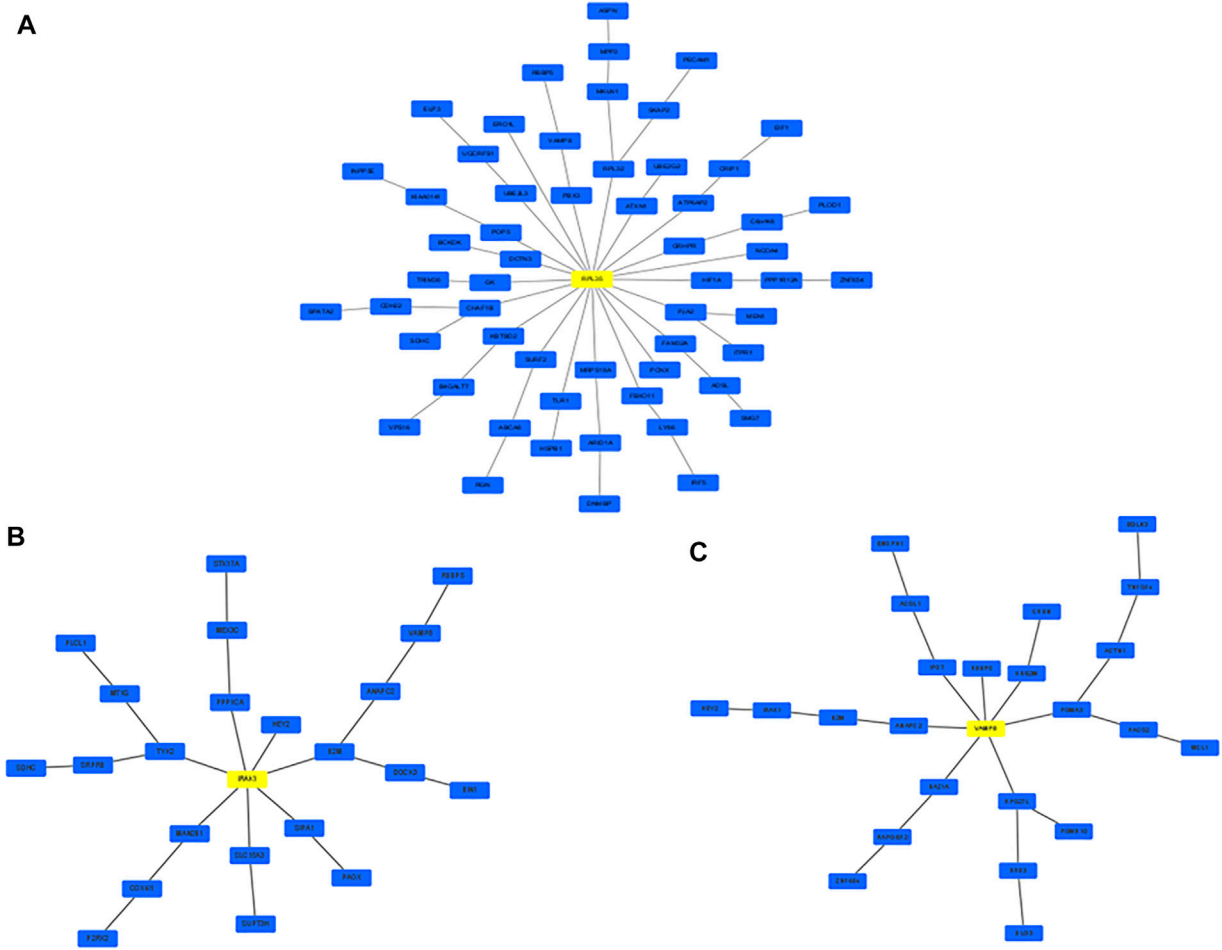
Network marker with the highest betweenness. **(A)** Based on MR; **(B,C)** based on MCMC.

Network markers were also identified by calculating the betweenness of each aging–disease shortest path; the top 10 markers are shown in Table 8. The network marker with the greatest betweenness (Munger et al., 2021) was RPL35 (Figure 5A; Table 8). RPL35 is an important component of the 60S ribosomal subunit, with a key role in mRNA translation and protein synthesis (Wu et al., 2021). Gene betweenness was also calculated according to the sensitivity analysis. The top markers and their betweenness values are shown in Table 9. According to this analysis, IRAK1 and VAMP8 were the top network markers (betweenness = 8; Figures 5B, C; Table 9). IRAK1 is a threonine/serine kinase that is related to the pathogenesis of atherosclerosis (Rana et al., 2016). It regulates lipid accumulation and prevents the formation of macrophage foam cells (Rana et al., 2016), as well as participating in multiple IL-1- and Toll-like receptors (TLR) driven signaling processes and in the regulation of levels of immune pro-inflammatory cytokines (Singer et al., 2018). VAMP8 is relevant to macrophage degranulation and secretion of TNF-*α* (Pushparaj et al., 2009), and regulates lysosome–autophagosome interaction and exocytosis (Jones et al., 2012; Chen et al., 2021b). Its phosphorylation regulates lipid metabolism in the liver (Huang et al., 2021). VAMP8 recruits NOX2 into phagosomes, causing lipid oxidation and membrane damage, and also mediates the transport of NOX2 to endosomes and phagosomes, thereby promoting the induction of the cytolytic T cell immune response (Dingjan et al., 2017). In short, the identification of network markers showed that the occurrence of atherosclerosis was closely related to inflammation and immunity homeostasis imbalance.

**TABLE 8:** Top 10 genes with the highest betweenness in the aging acceleration network.

Gene symbol	Betweenness	*p*-value
RPL35	21	0
IRAK1	20	0
VAMP8	19	0
LY86	17	0
MS4A3	17	0
IMP4	17	0
GDI1	16	0
PJA2	14	0
CLN3	14	0
C6orf48	14	0

**TABLE 9:** Top 10 genes with the highest betweenness in the aging acceleration network based on sensitivity analysis.

Gene symbol	Betweenness	*p*-value
IRAK1	8	0
VAMP8	8	0
RPL35	6	0
RPL18	6	0
PJA2	6	0
PARP6	6	0
JTB	5	0
SARS	5	0
HDGF	5	0
PPP1R12A	5	0

## Discussion

CVD has become a serious health problem, the incidence of which increases with age. It is well known that both aging and inflammation have vital roles in atherosclerosis, which is one of the key risk factors for CVD. However, the exact relationships between inflamm-aging and atherosclerosis and the role of immune homeostasis have to be studied more systematically. It is particularly important to elucidate the causal relationship between inflamm-aging and atherosclerosis, in order to determine the mechanisms underlying atherosclerosis and identify potential therapeutic targets. A causal analysis was used to identify the relationship between aging and atherosclerosis using MR. An ML approach was used owing to its great advantages in processing large amounts of data and generating prediction models with appropriate accuracies. Network analysis and sensitivity analysis were also performed to integrate multiple factors and identify relative risk markers, which will be conducive to more systematic study of atherosclerosis.

Our results showed that immune homeostasis is vital for the development of atherosclerosis. Disorders of the immune system can lead to atherosclerosis, and several characteristics related to immune homeostasis imbalance were identified in our study. For example, “myeloid leukocyte activation” (GO:0002274) and “regulation macrophage activation” (GO:0043030) involve the effects of inflammation on atherosclerosis. Several risk markers related to the immune response that have key roles in atherosclerosis were identified, including the top aging marker MMP8, disease markers KIR3DL1 and MAGEA6, network markers based on degree (RBM10, PJA2, and CMTM6; Table 4), and network markers based on betweenness (IRAK1 and VAMP8). Other potential markers including HEY2 (identified by sensitivity analysis) and PECAM1 (identified by MR and sensitivity analysis) and the top causal pairs BIRC2–PLOD1 and CRBN–MRPL40 also suggested a role of immunity in atherosclerosis. In brief, the abnormal immune response is a key characteristic of atherosclerosis (Karunakaran et al., 2015); our results further confirmed that immune homeostasis imbalance is crucial in atherosclerosis in the context of inflamm-aging.

Vascular homeostasis imbalance was also identified as a factor in atherosclerosis progression. It has been reported that dysfunction of endothelium and VSMCs, an early marker of atherosclerosis, can disrupt the balance between vasoconstriction and vasodilation, thereby promoting the inflammatory response and exacerbating atherosclerosis (Nkoenig et al., 2022; Alberts et al., 2002; Davignon and Ganz, 2004). The top inflammation marker (HSPA6) and the top network markers according to a degree (PXN and MAP3K3) in our results indicated an effect of endothelial cells and VSMCs. Dysfunction of endothelium contributes to the recruitment of inflammatory cells, and VASC proliferation is related to upregulation of pro-inflammatory cytokines (Alique et al., 2018). Consequently, there is a key link between inflammation and vascular homeostasis. The top enriched KEGG pathway was the “cytosolic DNA-sensing pathway” (Figure 4E), indicating the potential relevance of inflammation and vascular homeostasis. In addition, PECAM1 enables endothelial cells to respond to fluid shear stress and regulates inflammatory signaling pathways (Privratsky and Newman, 2014). In the causal pair BIRC2–PLOD1, BIRC2 promotes endothelial cell survival and blood vascular homeostasis *via* regulation of inflammatory/immune signaling pathways (Santoro et al., 2007; Samanta et al., 2020). PLOD1 encodes lysyl hydroxylase-1, which is essential for ECM maturation. ECM proteolytic fragments affect a variety of functions and properties of inflammatory and immune cells (Adairkirk and Senior, 2008); ECM was also relevant to the integrity and stability of VSMCs (Nkoenig et al., 2022). It has been reported that inflamm-aging can lead to changes in vascular structure and aging of VSMCs (Chi et al., 2019). In short, vascular homeostasis has a key role in atherosclerosis and inflamm-aging.

The role of oxidative stress was further investigated. Oxidative stress increases ROS generation and/or reduces the imbalance of the body’s innate antioxidant defense system and is thus a key influence on atherosclerosis (Kattoor et al., 2017). Our results provided further evidence of the association of oxidative stress with atherosclerosis, for instance, the top enriched BPs were “positive regulation of reactive oxygen species biosynthetic process” (GO:190,342) and “oxidative phosphorylation” (Table 4). Oxidative stress caused by excessive production of ROS has become the driven risk factor in atherosclerosis (Kattoor et al., 2017). The network marker (based on degree; Table 4) NADK is related to the production of NADPH. NADPH oxidase is the main source of ROS (Ganguly et al., 2021). ROS also has important roles in inflammatory response, apoptosis, cell growth, and changes in vascular tone as well as in the oxidation of LDL cholesterol (Kattoor et al., 2017). For example, ROS can cause physical damage to plaques *via* stimulating smooth muscle cell migration and collagen deposition, leading to endothelial dysfunction (Ganguly et al., 2021). In summary, the interaction between oxidative stress and vascular homeostasis is vital to atherosclerosis. The relationship between oxidative stress and aging was also explored. Oxidative stress is well known to have an important influence on aging. One of the aging markers identified by our MR approach was RPRM, a p53-inducible gene (Xu et al., 2012). TP53 regulates the expression of various genes involved in the maintenance of homeostasis, including cell cycle regulation and redox homeostasis (i.e., production of antioxidant enzymes) (Gambino et al., 2013; Beyfuss and Hood, 2018). Overall, these findings indicate that oxidative stress is a key factor in atherosclerosis in the context of inflamm-aging.

Nutritional imbalance (i.e., lipid and protein imbalance) has a vital role in atherosclerosis. Abnormal nutrient metabolism can affect the occurrence and development of atherosclerosis, and it has been suggested that atherosclerosis could be prevented and treated by adjusting the proportion of nutrients in the diet (Wei et al., 2021). For example, vesicles represent the main transport pathway for nutritional macromolecules (i.e., lipids and protein) and thus have a vital role in the establishment of the nutritional imbalance that is related to the development of atherosclerosis. The terms identified in our enrichment analyses, including “vesicle-mediated transport between endosomal compartments” (GO:0098927; Table 7) and “SNARE interactions in vesicular transport” (Table 6), confirmed this viewpoint. Furthermore, lipids have an important role in maintaining nutritional balance. Atherosclerosis is considered to be an inflammatory disease with chronic lipid accumulations and poor adaptability of arterial intima (So, 2018). Our sensitivity analysis identified CRBN, a negative regulator of AMPK *in vivo*, which is related to the metabolism of glucose and fat and is considered to be a regulator of body metabolism and energy homeostasis (Lee et al., 2013). We also identified IRAK1 and VAMP8 (using betweenness), which are related to the metabolism of lipids (Pushparaj et al., 2009; Singer et al., 2018). There is substantial evidence that lipids are related to atherosclerosis progression, as a high cholesterol level is a major risk factor in atherosclerosis and CVD, and fatty acid metabolism is closely related to endothelial function and protein homeostasis (Malekmohammad et al., 2021; Graham et al., 2021; Hasan and Fischer, 2021). Protein homeostasis is also relevant to macrophages in atherosclerosis (Koga et al., 2011). Our results demonstrated the role of protein as a nutrient in atherosclerosis. For instance, the top network marker (based on betweenness), RPL35, has a role in protein translation and endoplasmic reticulum docking (Wu et al., 2021). In addition, “aminoglycoside antibiotic metabolic process” (GO:0030647; Table 7) and “glycoside metabolic process” (GO:0016137; Table 7) further indicated a key role related to both lipids and proteins in atherosclerosis progression. As a result, the nutritional imbalance was identified as one of the driving factors behind atherosclerosis.

**FIGURE 6:**
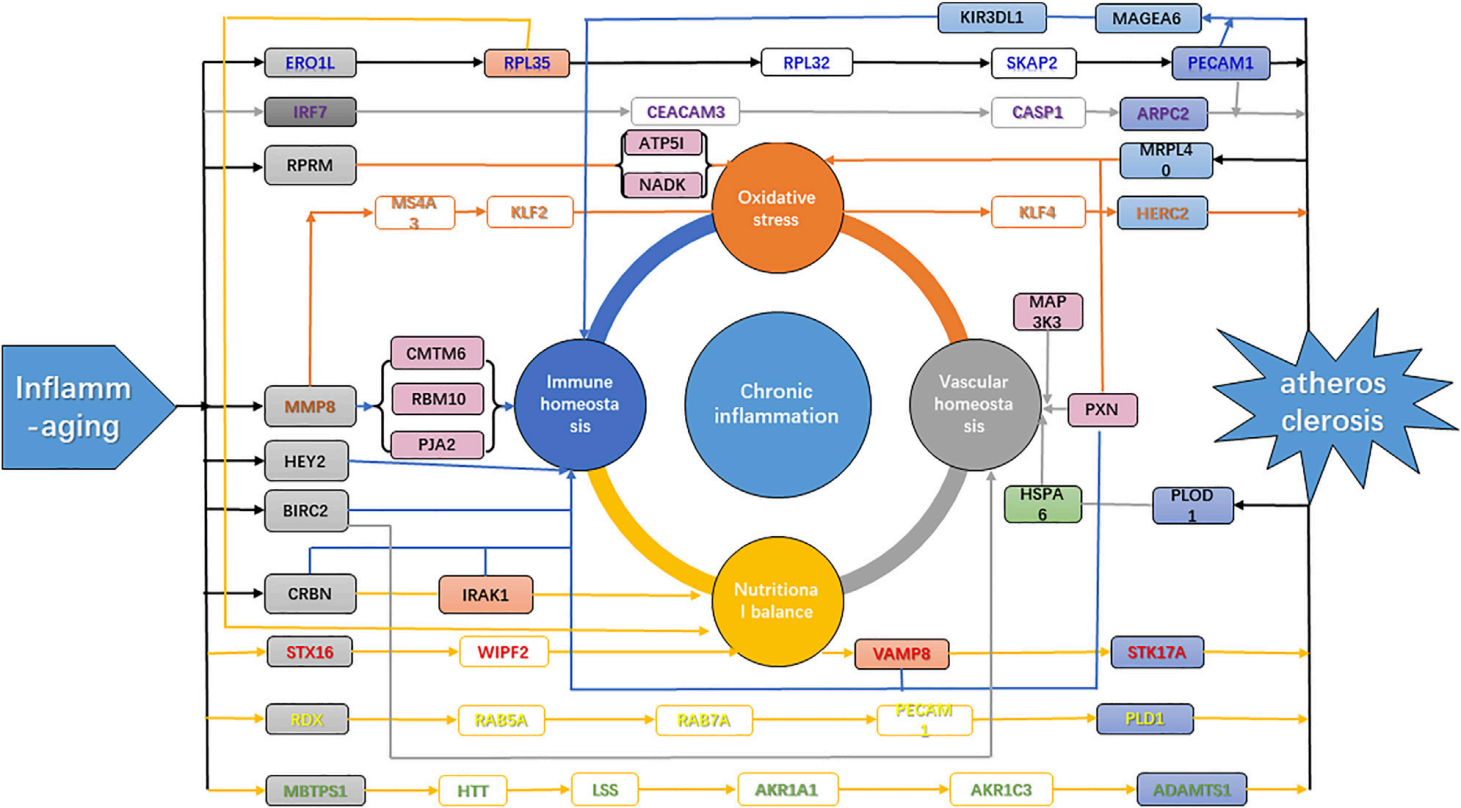
Mechanism of atherosclerosis induced by inflamm-aging. Gray genes, aging makers; blue genes, disease markers; green genes, inflammation markers; purple genes, markers with high degrees; orange genes, network nodes with high betweenness. Green arrow, inflammation; blue arrow, immune homeostasis; orange arrow, oxidative stress; gray arrow, vascular homeostasis; yellow arrow, nutritional balance.

In the theory of inflamm-aging, the aging process leads to a chronic progressive inflammatory state, involving both the activation of innate immunity and accelerated pro-inflammatory response. As a result, the dynamic balance between pro- and anti-inflammatory cytokines is disrupted, and atherosclerosis was induced by the resulting immune homeostasis imbalance (Kahn, 2021). Our results further confirmed the key role of inflamm-aging in atherosclerosis and also identified dysfunction of endothelium and vessels, oxidative stress, and nutritional imbalance as risk factors. Studies have shown interactions among inflammation, nutrition, and endothelial and vascular homeostasis as well as oxidative stress. For instance, lipid accumulation is involved in the production of pro-inflammatory cytokines (Malekmohammad et al., 2021) and inflammation can affect lipid metabolism in macrophages (Osada et al., 2017). Moreover, oxidized LDL (ox-LDL) can remain in blood vessels, inducing dysfunction of the endothelium, which further responds to inflammatory signals, with a crucial role in atherosclerosis development (Malekmohammad et al., 2021). In addition, increased ROS production promotes endothelial dysfunction, leading to remodeling, platelet aggregation, loss of vasodilation, inflammation, and smooth muscle cell growth (Higashi et al., 2014). In our analysis, the “ribosome” pathway was identified as the most enriched KEGG pathway (Figure 4A), which highlighted the interactions among nutrition, vessels, and oxidative stress during the aging process (Stein et al., 2022). In summary, our work integrated potential mechanisms involving the inflammatory response, nutrition imbalance, and vascular homeostasis imbalance as well as oxidative stress, consistent with and contributing further to the inflamm-aging theory (Figure 6).

## Conclusion

In this study, an ML method was used to model predictors of aging and disease and identify relative risk markers. The accelerated inflammatory pattern in atherosclerosis was verified by comparing inflamm-aging scores. The causal relationship between inflamm-aging and atherosclerosis was explored through MR, and then through sensitivity analysis, enrichment analysis, and network analysis; the results indicated that disorders of lipid homeostasis and dysfunction of endothelial cells and vascular homeostasis could promote the development of atherosclerosis. The role of oxidative stress was also confirmed. In summary, our work revealed that in the context of inflamm-aging, nutrition imbalance and vascular homeostasis, as well as oxidative stress, are risk factors that promote the development of atherosclerosis in a coordinated manner.

## Materials and Methods

### Data and Pre-Processing

All gene expression data were downloaded from the GEO database (https://www.ncbi.nlm.nih.gov/geo/) in NCBI, including the GSE7638, GSE9820, GSE12288, GSE20681, GSE22253, GSE37171, and GSE123696 datasets. These datasets were from seven different platforms: GPL571, GPL6255, GPL96, GPL4133, GPL6244, GPL570, and GPL15207. The pre-processing steps for obtaining gene expression profiles were as follows:

1) Age and disease information of each sample (disease was encoded as 1, healthy as 0) was obtained, and samples without age were deleted.
2) The gene expression matrix for each dataset was integrated by summarizing the probe number within the gene symbol.
3) The total data matrix was integrated, and the missing gene expression values were filled with values of 0.
4) Genes with zero expression values ≥ 30% were deleted.
5) Gene expression profiles were transformed by the logarithmic transformation method in containing outliers (>3 times of standard deviation or <−3 times of standard deviation).
6) Based on the mean and the standard deviation of gene expression for healthy aged individuals, z-score normalization was performed for both normal aged samples and atherosclerosis samples.
7) The singular value decomposition method was used to eliminate inter-sample variation based on the top three principal components in healthy aged individuals.
8) The z-score was then used to normalize all individuals based on the mean and standard deviation of healthy aged individuals.

As a result, a total of 926 samples were obtained, including 313 samples from healthy aged individuals (age >50 years, 210 training datasets +103 test datasets), 207 samples from healthy young individuals (age ≤50, 140 + 67), 340 samples from aged atherosclerosis patients (age >50, 235 + 105), and 66 samples from young atherosclerosis patients (age ≤50, 45 + 21), with a total of 11,313 gene symbols.

### Modeling the Improved Inflamm-Aging Predictor of Atherosclerosis

Gene expression profiles were divided into a training dataset and test dataset at a ratio close to 2:1, and the inflamm-aging prediction model was established by integrating aging, inflammation, and disease (atherosclerosis) markers. Aging and disease models were first constructed, respectively; then, an improved inflamm-aging model was developed by including inflamm-aging markers and adjusting the disease predictor (Supplementary Figure S5). The following steps were performed for the inflamm-aging model:

1) To summarize the interactions among key markers according to aging/disease, the gene expression profiles were transformed (or replaced) based on the results of our previous study (Wang et al., 2020b). The Pearson correlation coefficient was used to evaluate relevance and redundancy:

relevance=corr(gene_i.∗gene_j,phenotype), (1)

redundancy=corr(gene_i,gene_j), (2)

where the phenotype was set as 0 (control) versus 1 (disease) in the disease model, or as the transformation of the age in the aging model:

1/(1+exp(−(age−50)/50)). (3)

The interaction is summarized as follows:

$$\mathrm{int}eraction\_i=\sum_{j=1}^{\log2\left(n+1\right)}corr\left(gene\_i.*gene\_j,phenotype\right)*gene\_i.*gene\_j,$$ (4)

where only the first log2 (*n*+1) interactions for each gene were included.

2) The 11,313 gene profiles (training dataset) were sorted by the ReliefF algorithm.
3) The kNN (*k* = 5 with the cosine distance), SVM, NB, and ensemble learning (established 100 decision tree models) algorithms were used to select key markers (in the aging or disease model) based on the training dataset and to evaluate the performance on the test dataset, with the help of 10-fold cross-validation. The kNN (*k* = 5 with the cosine distance) algorithm was selected, and 441 aging markers and 390 candidate disease markers were identified in this step (Supplementary Tables S3, S4).
4) The inflamm-aging model was constructed by integrating the aging, inflammation, and disease markers as follows:
  ① 568 candidate inflammatory markers were obtained based on the GO BPs using the gene set enrichment analysis (GSEA) platform (http://software.broadinstitute.org/gsea/downloads.jsp, version 7.4, keyword “INFLAMMATORY”); then, aging markers or candidate disease markers overlapping with candidate inflammatory markers were removed.
  ② Aging markers and candidate disease markers were further filtered using the Kruskal–Wallis test (*p* < 0.05 and FDR < 0.2).
  ③ Inflamm-aging markers were identified by transforming selected inflammatory markers combined with the aging markers [also based on a previous study (Wang et al., 2020b) as in step (Lopez et al., 2021)], where an interaction was retained if *p* < 0.05 and FDR < 0.2 using the Kruskal–Wallis test.
  ④ Disease markers were also transformed based on the inflammatory markers.
  ⑤ The kNN method, ReliefF algorithm, and 10-fold cross-validation were used to reselect the optimized integrative disease markers, where some of the top candidate 500 disease markers were replaced by disease markers related to inflamm-aging in step ④. Therefore, the improved inflamm-aging predictor contained 116 aging markers, 186 inflammatory markers, and 316 disease markers (Supplementary Table S5).
5) The traditional aging or disease predictor using original gene datasets was used as a comparator to evaluate the prediction ability of the improved inflamm-aging model (also using kNN, ReliefF, and 10-fold cross-validation with the same details; results in Supplementary Table S6, S7).
6) Other classifiers including SVM, NB, and the ensemble algorithm were also used for comparison of their prediction abilities.

### Calculating the Inflamm-Aging Score

The steps to calculate the inflamm-aging score for each sample were as follows:

1) The distance between healthy young and old neighbors was calculated using the original inflamm-aging score based on the inflammatory markers in the inflamm-aging model; the cosine distance was also used:

$$\inf lamm-aging\_score=\sum_{i=1}^{9}dis\tan ce\_in\_young-\sum_{i=1}^{9}dis\tan ce\_in\_old.$$ (5)

2) The original score was adjusted by age:

adjust_score=score−b∗transformated_age, (6)

where b is the regression coefficient for an aging score, and the transformed age is calculated as follows:

$$\text{tanh}\left(\frac{age-50}{50}\right).$$. (7)

3) Both the original and adjusted inflamm-aging scores were used to test the accelerated pattern in atherosclerosis based on the Kruskal–Wallis test.
4) The atherosclerosis (disease) score was calculated based on the identified disease markers (with the same details as for the inflamm-aging score) in the improved inflamm-aging model for further (sensitivity/network) analysis.

### Causality Analysis Based on MR

MR is a statistical method used to evaluate the causal relationship between risk factors and results based on observed data (Burgess et al., 2019; Burgess et al., 2020). The causal relationships among the instrumental variable, the risk factor, and the outcome variable were evaluated as follows.

1) There was a correlation between the instrumental variable and the risk factor.
2) There was no correlation between the instrumental variable and the confounding factor.
3) There was no correlation between the instrumental variable and the outcome variable after deleting the effect from the risk factor.

In this work, aging markers were used as instrumental variables, inflammatory markers were used as risk factors, and inflamm-aging markers were used as outcome variables to explore the causal relationship between inflamm-aging and atherosclerosis. The steps were as follows:

1) The training dataset was divided into three groups: low inflammation in health (“inflammation− atherosclerosis-”), high inflammation in health (“inflammation+ atherosclerosis−“), and low inflammation in disease (“inflammation+ atherosclerosis+”).
2) For each group, the Pearson correlation coefficient between each aging marker and the inflamm-aging score was calculated to evaluate the correlation between the instrument variable and the risk factor. The relationship was retained if the correlation for “inflammation− atherosclerosis− was between those of “inflammation+ atherosclerosis−” and “inflammation+ atherosclerosis+” (meaning that the atherosclerosis was triggered by the inflammation).
3) To filter out the correlation between the instrumental variable and confounding factors, a permutation test was performed by generating the simulated inflamm-aging score from the same number of randomly selected markers; this process was repeated 1,000 times, then the *p*-value was calculated as the proportion of occurrence times (larger than the real difference) of the absolute difference between “inflammation+ atherosclerosis−” and “inflammation+ atherosclerosis+” in 1,000 permutations. The relationship between each aging marker and the inflamm-aging score was retained if the permutation *p* < 0.05.
4) If the gene was within both the aging marker set and the disease marker set, then it was removed.
5) For each group, the Pearson correlation coefficient between each aging marker and disease marker was calculated to evaluate the correlation between the instrument variable and the outcome variable; and the partial Pearson correlation coefficient between each aging marker and disease marker based on the inflamm-aging score was calculated to evaluate the correlation between the instrument variable and the outcome variable without the background of the risk factor. The causal relationship between each aging marker and disease marker was retained if the difference between the correlation and the partial correlation for “inflammation− atherosclerosis− was between those for “inflammation+ atherosclerosis−” and “inflammation+ atherosclerosis+” (meaning that the atherosclerosis was triggered by the inflammation).
6) To filter out the potential correlation between confounding factors and the outcome variable, a permutation test was performed by generating the simulated aging marker and disease marker from the same number of randomly selected markers, repeated 1,000 times; then, the permutation P-value was calculated using the same details as in step (Pahwa and Jialal, 2021).
7) To filter out potential weak instrumental variables, the aging markers were further analyzed by evaluating the correlation between the inflamm-aging score and each aging marker, using the result of the point multiplication between the inflamm-aging score and each aging marker; then, the residual of the inflamm-aging score was tested between the atherosclerosis and control subgroup, using the Kruskal–Wallis test (*p* < 0.05 and FDR < 0.2).

residual=inflamm−aging_score−b∗(inflamm−aging_score.∗aging_marker), (8)

where *b* is the regression coefficient.

8) To filter out the effect of horizontal pleiotropy, the aging–disease causal relationship was further examined by comparing the correlation between each aging and disease marker, through the inflamm-aging score or otherwise. Steps ①–③ were used to calculate the correlations between instrumental variables and outcome variables without the background of the risk factor, and step ④ was used to calculate the correlations between instrumental variables and outcome variables without the context of the risk factor.

① The residual of each disease marker (“residual A”) is calculated based on the inflamm-aging score:

$$\text{residual}\_A=disease\_mar\ker-b_{1}*\left(disease\_mar\ker.*\inf lamm-aging\_Score\right),$$ (9)

where *b1* is the regression coefficient.

② The residual of each aging marker (“residual B”) is calculated based on the inflamm-aging score:

$$\text{residual}\_B=disease\_mar\ker-b_{2}*\left(disease\_mar\ker.*aging\_mar\ker\right),$$ (10)

where *b*_*2*_ is the regression coefficient.

③ The aforementioned two residuals were further compared, and the residual of the disease marker is calculated (as “residual C”):

$$\text{residual}\_C=residual\_A-b_{3}*residua-\_B,$$ (11)

where *b*_*3*_ is the regression coefficient.

④ The residual of the disease marker (“residual D”) was calculated based on the aging marker.

⑤ The difference (between “residual C” and “residual D”) was tested between the atherosclerosis and control subgroups, using the Kruskal–Wallis test (P < 0.05 and FDR<0.2).

Finally, the causal relationship between the aging marker and disease marker (through the inflamm-aging score) was retained. Thus, 1,340 aging–disease pairs were identified as causal relationships, including 116 aging markers and 312 inflamm-aging markers.

### Constructing the Inflamm-Aging Differential Network

In order to further reveal the relationship between inflamm-aging and atherosclerosis, an inflamm-aging differential network was constructed by the following steps:

1) To compare the relationship of each gene pair in the context of inflamm-aging, both the Pearson correlation coefficient for each pair of genes and the partial correlation coefficient based on the inflamm-aging score were calculated based on the atherosclerosis and control groups, respectively.
2) The Benjamini–Hochberg FDR method was used to adjust the P-values of the correlation coefficient and the partial correlation coefficient.
3) The differences in the correlations and partial correlations between the atherosclerosis and control groups were calculated.
4) The edge between each gene pair was retained if the signs of the difference in values for the correlation and partial correlation were different, and values of *p* < 0.05 and FDR<0.1 were obtained in step (Pahwa and Jialal, 2021).
5) The scale-free characteristics of the inflamm-aging acceleration differential networks were verified by the power-law distribution.
6) The shortest path between each pair of aging and inflamm-aging markers was selected based on each aging acceleration differential network using the Djikstra algorithm.
7) The network constructed based on the training data was used for further analysis (identifying shortest paths, exploring biological functions between aging and atherosclerosis, etc.), and the network constructed based on the test data was used to validate the training network.

### Global Sensitivity Analysis Using the MCMC Method

In order to further explore the immune homeostasis imbalance in atherosclerosis in the context of inflamm-aging, an overall sensitivity analysis based on the MCMC method was carried out, where the aging–disease pairs identified by MR were used as candidate relationships to be further evaluated. The MCMC method is used for sampling from certain posterior distributions following a given probabilistic background in high-dimensional space. The key step in MCMC is to construct a Markov chain whose equilibrium distribution equals the target probability distribution. The steps are as follows.

1) Construct a transition kernel of an ergodic Markov chain. In this study, the prior distribution for each parameter was the normal distribution based on all the identified aging inflammation and inflamm-aging markers for each group (“inflammation− atherosclerosis−”, “inflammation+ atherosclerosis−, and “inflammation+ atherosclerosis+”).
2) Simulate the chain until it reaches equilibrium. The Metropolis–Hastings sampling method is used to determine whether the new sample (θ*) is acceptable based on the *α* value:

$$\alpha =\frac{P\left(\theta^{*}|X\right)*q\left(\theta^{n}\to \theta^{*}\right)}{P\left(\theta^{n}|X\right)*q\left(\theta^{n}\to \theta^{*}\right)},$$ (12)

where *p*(*θ* ^*n*^ *|X*) and *p*(*θ* ^*^ *|X*) are the posterior probabilities of the *n*th accepted sample and the new sample, *q*(*θ* ^*n*^ *→θ* ^*^) is the transition probability from the *n*th accepted sample to the new sample, and *q*(*θ* ^*^ *→θ* ^*n*^) is the transition probability from the new sample to the *n*th accepted sample.

In this work, the inflamm-aging score and disease score were used to evaluate the simulated samples, with 1,000 random samples used as candidate samples for each group.

3) Perform global sensitivity analysis. In this study, the distribution (ratio) of each aging–disease pair is calculated as follows:

∑sign(aging_marker.∗disease_marker). (13)

In addition, if the value for “inflammation− atherosclerosis− was between those of “inflammation+ atherosclerosis− and “inflammation+ atherosclerosis+”, then the aging–disease pair was identified as a sensitive relationship for further network analysis. As a result, 442 aging–disease pairs were identified as causal relationships, including 93 aging markers and 81 disease markers.

### Enrichment Analysis

Gene functions with significant correlations were further explored through enrichment analysis of the shortest paths. GO terms and KEGG pathways were identified using the GSEA platform (http://software.broadinstitute.org/gsea/downloads.jsp, version 7.4). The hypergeometric distribution was used to test the enrichment of GO BP terms and KEGG pathways using the following formula:

$$P\left(X\geq x\right)=1-\sum_{k=0}^{x-1}\frac{C_{M}^{k}*C_{N-M}^{n-k}}{C_{N}^{k}},$$ (14)

where *N* is the total number of genes in the gene set, *M* is the number of known genes (KEGG pathways or BP terms), *n* is the number of genes identified in each shortest path, and *k* is the number of common genes between known genes and candidate genes identified in each aging–disease shortest path. The *p*-value for each path was controlled using the Benjamini–Hochberg method. Finally, the paths with *p* < 0.05 and FDR<01 were retained. The enrichment score is calculated as follows:

$$score=\sum_{FDR<0.1}\left(1-FDR_{i}\right).$$ (15)

### Identifying Network Markers

The subnetwork with the shortest pathways among the selected aging–disease pairs was constructed (after MR or MCMC), and genes in the subnetwork were sorted by their betweenness in descending order. To test whether the top betweenness genes were hubs in the background network, we ran a permutation to count the occurrences of the top genes in the shortest paths between randomly selected genes (containing the same numbers of aging–disease pairs, 1,340 in MR or 442 in MCMC) when they had greater betweennesses than those in our study. We repeated this process 1,000 times, and the *p*-value was calculated as the proportion of occurrences of the top betweenness genes in 1,000 permutations.

## Data Availability

The original contributions presented in the study are included in the article/Supplementary Material; further inquiries can be directed to the corresponding author.

## Supplementary Material

Supplementary Figure S1 ML results for control methods. **(A, B)** Traditional aging predictor. **(C,D)** Traditional atherosclerosis predictor. **(C,D)** Disease predictor from our previous study. **(A,C,E)** Learning curve for the training dataset. **(B,D,F)** ROC curve for the test dataset.

Supplementary Figure S2 PCA plots of the inflamm-aging model based on **(A)** the inflamm-aging score and disease risk score; and **(B)** the first two principal components. Blue circle, normal test set; blue ‘+’, normal training set; red circle, atherosclerosis test set; red ‘+’, atherosclerosis training set.

Supplementary Figure S3 Degree distribution of the inflamm-aging differential network.

Supplementary Figure S4 Heatmap of the bipartite graph between aging markers and disease markers based on MCMC. The *x*-axis represents disease markers; the *Y-axis* represents aging markers.

Supplementary Figure S5 Workflow of the improved inflamm-aging model.

Supplementary Table S1 Detailed datasets used in this work.

Supplementary Table S2 Gene symbols used in this work.

Supplementary Table S3 Selected aging markers from our previous study, with their ReliefF weights.

Supplementary Table S4 Selected disease markers from our previous study, with their ReliefF weights.

Supplementary Table S5 Selected aging, inflammatory, and disease markers in the improved inflamm-aging model, with their P-values, FDRs, and ReliefF weights.

Supplementary Table S6 Selected aging markers obtained by the traditional method, with their ReliefF weights.

Supplementary Table S7 Selected disease markers obtained by the traditional method, with their ReliefF weights.

## Abbreviations

AUC, area under the ROC curve; BP, biological process; CVD, cardiovascular disease; ECM, extracellular matrix; FDR, false discovery rate; GEO, Gene Expression Omnibus; GO, Gene Ontology; GSEA, gene set enrichment analysis; KEGG, Kyoto Encyclopedia of Genes and Genomes; KIR, killer immunoglobulin-like receptors; kNN, k-nearest neighbors; MCMC, Markov chain Monte Carlo; ML, machine learning; MMP, matrix metalloproteinase; MR, Mendelian randomization; NB, naive Bayes; OXPHOS, oxidative phosphorylation; PCA, principal components analysis; ROC, receiver operating characteristic; ROS, reactive oxygen species; SVM, support vector machine; TLR, toll-like receptors; VSMC, vascular smooth muscle cells.

## References

[B1] Adairkirk T. Senior R. (2008). Fragments of Extracellular Matrix as Mediators of Inflammation. Int. J. Biochem. Cell Biol. 40 (6-7), 1101–1110. 10.1016/j.biocel.2007.12.005 18243041 PMC2478752

[B2] Alalawi H. H. Alsuwat M. S. (2021). Detection of Cardiovascular Disease Using Machine Learning Classification Models. IJERT 07, 14.

[B3] Alber M. Buganza Tepole A. Cannon W. R. De S. Dura-Bernal S. Garikipati K. (2019). Integrating Machine Learning and Multiscale Modeling-Perspectives, Challenges, and Opportunities in the Biological, Biomedical, and Behavioral Sciences. npj Digit. Med. 2, 115. 10.1038/s41746-019-0193-y 31799423 PMC6877584

[B4] Alberts B. Johnson A. Lewis J. Raff M. Roberts K. Walter P. (2002). Molecular Biology of the Cell. 4th edition. New York: Garland Science.

[B5] Alique M. Ramírez-Carracedo R. Bodega G. Carracedo J. Ramírez R. (2018). Senescent Microvesicles: A Novel Advance in Molecular Mechanisms of Atherosclerotic Calcification. Int. J. Mol. Sci. 19 (7), 2003. 10.3390/ijms19072003 PMC6073566 29987251

[B6] Bennett M. R. Sinha S. Owens G. K. (2016). Vascular Smooth Muscle Cells in Atherosclerosis. Circ. Res. 118 (4), 692–702. 10.1161/CIRCRESAHA.115.306361 26892967 PMC4762053

[B7] Bergheanu S. C. Bodde M. C. Jukema J. W. (2017). Pathophysiology and Treatment of Atherosclerosis. Neth Heart J. 25 (4), 231–242. 10.1007/s12471-017-0959-2 28194698 PMC5355390

[B8] Beyfuss K. Hood D. A. (2018). A Systematic Review of P53 Regulation of Oxidative Stress in Skeletal Muscle. Redox Rep. 23 (1), 100–117. 10.1080/13510002.2017.1416773 29298131 PMC6748683

[B9] Blachier F. Andriamihaja M. Blais A. (2020). Sulfur-Containing Amino Acids and Lipid Metabolism. J. Nutr. 150 (Suppl. 1), 2524S–2531S. 10.1093/jn/nxaa243 33000164

[B10] Bonifácio V. D. B. Pereira S. A. Serpa J. Vicente J. B. (2021). Cysteine Metabolic Circuitries: Druggable Targets in Cancer. Br. J. Cancer 124, 862–879. 10.1038/s41416-020-01156-1 33223534 PMC7921671

[B11] Burgess S. Davey Smith G. Davies N. M. Dudbridge F. Gill D. Glymour M. M. (2019). Guidelines for Performing Mendelian Randomization Investigations. Wellcome Open Res. 4, 186. 10.12688/wellcomeopenres.15555.1 32760811 PMC7384151

[B12] Burgess S. Foley C. N. Allara E. Staley J. R. Howson J. M. M. (2020). A Robust and Efficient Method for Mendelian Randomization with Hundreds of Genetic Variants. Nat. Commun. 11, 376. 10.1038/s41467-019-14156-4 31953392 PMC6969055

[B13] Cao Y. Di X. Zhang Q. Li R. Wang K. (2021). RBM10 Regulates Tumor Apoptosis, Proliferation, and Metastasis. Front. Oncol. 11, 603932. 10.3389/fonc.2021.603932 33718153 PMC7943715

[B14] Chandra R. K. (1997). Nutrition and the Immune System: an Introduction. Am. J. Clin. Nutr. 66 (2), 460S–463S. 10.1093/ajcn/66.2.460S 9250133

[B15] Chen P.-L. Huang K.-T. Cheng C.-Y. Li J.-C. Chan H.-Y. Lin T.-Y. (2020). Vesicular Transport Mediates the Uptake of Cytoplasmic Proteins into Mitochondria in *Drosophila melanogaster* . Nat. Commun. 11, 2592. 10.1038/s41467-020-16335-0 32444642 PMC7244744

[B16] Chen Q. Hao M. Wang L. Li L. Chen Y. Shao X. (2021). Prefused Lysosomes Cluster on Autophagosomes Regulated by VAMP8. Cell Death Dis. 12, 939. 10.1038/s41419-021-04243-0 34645799 PMC8514493

[B17] Chen Y. Zhao H. Xiao Y. Shen P. Tan L. Zhang S. (2021). Pan-cancer Analysis Reveals an Immunological Role and Prognostic Potential of PXN in Human Cancer. Aging 13 (12), 16248–16266. 10.18632/aging.203154 34135128 PMC8266322

[B18] Chi C. Li D. J. Jiang Y. J. Tong J. Fu H. Wu Y. H. (2019). Vascular Smooth Muscle Cell Senescence and Age-Related Diseases: State of the Art. Biochim. Biophys. Acta Mol. Basis Dis. 1865, 1810–1821. 10.1016/j.bbadis.2018.08.015 31109451

[B19] Childs B. G. Gluscevic M. Baker D. J. Laberge R.-M. Marquess D. Dananberg J. (2017). Senescent Cells: an Emerging Target for Diseases of Ageing. Nat. Rev. Drug. Discov. 16, 718–735. 10.1038/nrd.2017.116 28729727 PMC5942225

[B20] Chothani S. Schäfer S. Adami E. Viswanathan S. Widjaja A. A. Langley S. R. (2019). Widespread Translational Control of Fibrosis in the Human Heart by RNA-Binding Proteins. Circulation 140 (11), 937–951. 10.1161/CIRCULATIONAHA.119.039596 31284728 PMC6749977

[B21] Covarrubias A. J. Perrone R. Grozio A. Verdin E. (2021). NAD+ Metabolism and its Roles in Cellular Processes during Ageing. Nat. Rev. Mol. Cell Biol. 22 (2), 119–141. 10.1038/s41580-020-00313-x 33353981 PMC7963035

[B22] Critchley B. J. Isalan M. Mielcarek M. (2018). Neuro-Cardio Mechanisms in Huntington's Disease and Other Neurodegenerative Disorders. Front. Physiol. 9. 10.3389/fphys.2018.00559 PMC5974550 29875678

[B23] Davignon J. Ganz P. (2004). Role of Endothelial Dysfunction in Atherosclerosis. Circulation 109 (23 Suppl. 1), III27–32. 10.1161/01.CIR.0000131515.03336.f8 15198963

[B24] De Stefano A. Caporali S. Di Daniele N. Rovella V. Cardillo C. Schinzari F. (2021). Anti-Inflammatory and Proliferative Properties of Luteolin-7-O-Glucoside. Int. J. Mol. Sci. 22 (3), 1321. 10.3390/ijms22031321 33525692 PMC7865871

[B25] Deshpande V. Sharma A. Mukhopadhyay R. Thota L. N. R. Ghatge M. Vangala R. K. (2016). Understanding the Progression of Atherosclerosis through Gene Profiling and Co-expression Network Analysis in Apob tm2Sgy Ldlr tm1Her Double Knockout Mice. Genomics 107 (6), 239–247. 10.1016/j.ygeno.2016.04.007 27133569

[B26] Dingjan I. Paardekooper L. M. Verboogen D. R. J. von Mollard G. F. Ter Beest M. van den Bogaart G. (2017). VAMP8-mediated NOX2 Recruitment to Endosomes Is Necessary for Antigen Release. Eur. J. Cell Biol. 96 (7), 705–714. 10.1016/j.ejcb.2017.06.007 28688576 PMC5641923

[B27] El Assar M. Angulo J. Vallejo S. Peiró C. Sánchez-Ferrer C. F. Rodríguez-Mañas L. (2012). Mechanisms Involved in the Aging-Induced Vascular Dysfunction. Front. Physio. 3, 132. 10.3389/fphys.2012.00132 PMC3361078 22783194

[B28] Fabris F. Magalhães J. P. d. Freitas A. A. (2017). A Review of Supervised Machine Learning Applied to Ageing Research. Biogerontology 18 (2), 171–188. 10.1007/s10522-017-9683-y 28265788 PMC5350215

[B29] Figueroa R. J. Carrasco-Avino G. Wichmann I. A. Lange M. Owen G. I. Siekmann A. F. (2017). Reprimo Tissue-specific Expression Pattern Is Conserved between Zebrafish and Human. PLoS One 12 (5), e0178274. 10.1371/journal.pone.0178274 28562620 PMC5451059

[B30] Fogoros R. N. (2020). “An Overview of Atherosclerosis,” in Verywell Health. Editor Ali Y. S..

[B31] Fuente M. Miquel J. (2009). An Update of the Oxidation-Inflammation Theory of Aging: the Involvement of the Immune System in Oxi-Inflamm-Aging. Cpd 15 (26), 3003–3026. 10.2174/138161209789058110 19754376

[B32] Gambino V. De Michele G. Venezia O. Migliaccio P. Dall'Olio V. Bernard L. (2013). Oxidative Stress Activates a Specific P53 Transcriptional Response that Regulates Cellular Senescence and Aging. Aging Cell 12, 3435–3445. 10.1111/acel.12060 PMC3709138 23448364

[B33] Ganguly U. Kaur U. Chakrabarti S. S. Sharma P. Agrawal B. K. Saso L. (2021). Oxidative Stress, Neuroinflammation, and NADPH Oxidase: Implications in the Pathogenesis and Treatment of Alzheimer's Disease. Oxidative Med. Cell. Longev. 2021, 1–19. 10.1155/2021/7086512 PMC8068554 33953837

[B34] Gonzalez L. S. Spencer J. P. (1998). Aminoglycosides: A Practical Review. Am. Fam. Physician 58 (8), 1811–1820. 9835856

[B35] Graham S. E. Clarke S. L. Wu K. H. H. Kanoni S. Zajac G. J. M. Ramdas S. (2021). The Power of Genetic Diversity in Genome-wide Association Studies of Lipids. Nature 600, 675. 10.1038/s41586-021-04064-3 34887591 PMC8730582

[B36] Guan X. Zhang C. Zhao J. Sun G. Song Q. Jia W. (2018). CMTM6 Overexpression Is Associated with Molecular and Clinical Characteristics of Malignancy and Predicts Poor Prognosis in Gliomas. EbioMedicine 35, 233–243. 10.1016/j.ebiom.2018.08.012 30131308 PMC6156716

[B37] Harry B. L. Sanders J. M. Feaver R. E. Lansey M. Deem T. L. Zarbock A. (2008). Endothelial Cell PECAM-1 Promotes Atherosclerotic Lesions in Areas of Disturbed Flow in ApoE-Deficient Mice. Atvb 28, 2003–2008. 10.1161/atvbaha.108.164707 PMC2651147 18688018

[B38] Hasan S. S. Fischer A. (2021). The Endothelium: An Active Regulator of Lipid and Glucose Homeostasis. Trends Cell Biol. 31 (1), 37–49. 10.1016/j.tcb.2020.10.003 33129632

[B39] Higashi Y. Maruhashi T. Noma K. Kihara Y. (2014). Oxidative Stress and Endothelial Dysfunction: Clinical Evidence and Therapeutic Implications. Trends Cardiovasc Med. 24 (4), 165–169. 10.1016/j.tcm.2013.12.001 24373981

[B40] Hosseini Z. Marinello M. Decker C. Sansbury B. E. Sadhu S. Gerlach B. D. (2021). Resolvin D1 Enhances Necroptotic Cell Clearance through Promoting Macrophage Fatty Acid Oxidation and Oxidative Phosphorylation. Atvb 41, 1062–1075. 10.1161/atvbaha.120.315758 PMC8174560 33472399

[B41] Hu Y.-B. Dammer E. B. Ren R.-J. Wang G. (2015). The Endosomal-Lysosomal System: from Acidification and Cargo Sorting to Neurodegeneration. Transl. Neurodegener. 4, 18. 10.1186/s40035-015-0041-1 26448863 PMC4596472

[B42] Huang H. Ouyang Q. Zhu M. Yu H. Mei K. Liu R. (2021). mTOR-Mediated Phosphorylation of VAMP8 and SCFD1 Regulates Autophagosome Maturation. Nat. Commun. 12, 6622. 10.1038/s41467-021-26824-5 34785650 PMC8595342

[B43] Hubler M. J. Kennedy A. J. (2016). Role of Lipids in the Metabolism and Activation of Immune Cells. J. Nutr. Biochem. 34, 1–7. 10.1016/j.jnutbio.2015.11.002 27424223 PMC5694687

[B44] Jeon S. Yoon Y.-S. Kim H. K. Han J. Lee K. M. Seol J. E. (2021). Ablation of CRBN Induces Loss of Type I Collagen and SCH in Mouse Skin by Fibroblast Senescence via the P38 MAPK Pathway. Aging 13 (5), 6406–6419. 10.18632/aging.202744 33658395 PMC7993720

[B45] Jia F. Wu Q. Wang Z. Zhang M. Yuan S. Che Y. (2021). BOP1 Knockdown Attenuates Neointimal Hyperplasia by Activating P53 and Inhibiting Nascent Protein Synthesis. Oxidative Med. Cell. Longev. 2021, 1–20. 10.1155/2021/5986260 PMC7826231 33510838

[B46] Jia L. Kaur J. Stuart R. A. (2009). Mapping of the *Saccharomyces cerevisiae* Oxa1-Mitochondrial Ribosome Interface and Identification of MrpL40, a Ribosomal Protein in Close Proximity to Oxa1 and Critical for Oxidative Phosphorylation Complex Assembly. Eukaryot. Cell 8 (11), 1792–1802. 10.1128/ec.00219-09 19783770 PMC2772399

[B47] Jones L. C. Moussa L. Fulcher M. L. Zhu Y. Hudson E. J. O'Neal W. K. (2012). VAMP8 Is a Vesicle SNARE that Regulates Mucin Secretion in Airway Goblet Cells. J. Physiol. 590 (Pt 3), 545–562. 10.1113/jphysiol.2011.222091 22144578 PMC3379700

[B48] Kahn J. (2021). Atherosclerosis and Aging — Insights into the Role of the Endothelial Glycocalyx in Cardiovascular Health. Available at: https://www.todaysgeriatricmedicine.com/news/ex_052419.shtml .

[B49] Karunakaran D. Thrush A. B. Nguyen M.-A. Richards L. Geoffrion M. Singaravelu R. (2015). Macrophage Mitochondrial Energy Status Regulates Cholesterol Efflux and Is Enhanced by Anti-miR33 in Atherosclerosis. Circ. Res. 117 (3), 266–278. 10.1161/CIRCRESAHA.117.305624 26002865 PMC4578799

[B50] Kattoor A. J. Pothineni N. V. K. Palagiri D. Mehta J. L. (2017). Oxidative Stress in Atherosclerosis. Curr. Atheroscler. Rep. 19, 42. 10.1007/s11883-017-0678-6 28921056

[B51] Keesey R. E. Powley T. L. (2008). Body Energy Homeostasis. Appetite 51 (3), 442–445. 10.1016/j.appet.2008.06.009 18647629 PMC2605663

[B52] Kiechl S. Willeit J. Rungger G. Egger G. Oberhollenzer F. Bonora E. (1998). Alcohol Consumption and Atherosclerosis: What Is the Relation? Stroke 29 (5), 900–907. 10.1161/01.str.29.5.900 9596232

[B53] Klemm S. Ruland J. (2006). Inflammatory Signal Transduction from the FcεRI to NF-Κb. Immunobiology 211 (10), 815–820. 10.1016/j.imbio.2006.07.001 17113919

[B54] Koga H. Kaushik S. Cuervo A. M. (2011). Protein Homeostasis and Aging: the Importance of Exquisite Quality Control. Ageing Res. Rev. 10 (2), 205–215. 10.1016/j.arr.2010.02.001 20152936 PMC2888802

[B55] Konkoth A. Saraswat R. Dubrou C. Sabatier F. Leroyer A. S. Lacroix R. (2021). Multifaceted Role of Extracellular Vesicles in Atherosclerosis. Atherosclerosis 319, 121–131. 10.1016/j.atherosclerosis.2020.11.006 33261815

[B56] Krause K. M. Serio A. W. Kane T. R. Connolly L. E. (2016). Aminoglycosides: An Overview. Cold Spring Harb. Perspect. Med. 6 (6), a027029. 10.1101/cshperspect.a027029 27252397 PMC4888811

[B57] Kumar A. Palfrey H. A. Pathak R. Kadowitz P. J. Gettys T. W. Murthy S. N. (2017). The Metabolism and Significance of Homocysteine in Nutrition and Health. Nutr. Metab. (Lond) 14, 78. 10.1186/s12986-017-0233-z 29299040 PMC5741875

[B58] Kurek J. M. Król E. Krejpcio Z. (2021). Steviol Glycosides Supplementation Affects Lipid Metabolism in High-Fat Fed STZ-Induced Diabetic Rats. Nutrients 13 (1), 112. 10.3390/nu13010112 PMC7823366 33396905

[B59] Lan Y. Lou J. Hu J. Yu Z. Lyu W. Zhang B. (2020). Downregulation of SNRPG Induces Cell Cycle Arrest and Sensitizes Human Glioblastoma Cells to Temozolomide by Targeting Myc through a P53-dependent Signaling Pathway. Cancer Biol. Med. 17 (1), 112–131. 10.20892/j.issn.2095-3941.2019.0164 32296580 PMC7142844

[B60] Lathe R. Sapronova A. Kotelevtsev Y. (2014). Atherosclerosis and Alzheimer - Diseases with a Common Cause? Inflammation, Oxysterols, Vasculature. BMC Geriatr. 14, 36. 10.1186/1471-2318-14-36 24656052 PMC3994432

[B61] Lavin Plaza B. Phinikaridou A. Andia M. E. Potter M. Lorrio S. Rashid I. (2020). Sustained Focal Vascular Inflammation Accelerates Atherosclerosis in Remote Arteries. Atvb 40 (9), 2159–2170. 10.1161/atvbaha.120.314387 PMC7447189 32673527

[B62] Laxton R. C. Hu Y. Duchene J. Zhang F. Zhang Z. Leung K.-Y. (2009). A Role of Matrix Metalloproteinase-8 in Atherosclerosis. Circulation Res. 105 (9), 921–929. 10.1161/CIRCRESAHA.109.200279 19745165 PMC2853782

[B63] Lee K. M. Yang S.-J. Kim Y. D. Choi Y. D. Nam J. H. Choi C. S. (2013). Disruption of the Cereblon Gene Enhances Hepatic AMPK Activity and Prevents High-Fat Diet-Induced Obesity and Insulin Resistance in Mice. Diabetes 62 (6), 1855–1864. 10.2337/db12-1030 23349485 PMC3661653

[B64] Li J. Ryan S. K. Deboer E. Cook K. Fitzgerald S. Lachman H. M. (2019). Mitochondrial Deficits in Human iPSC-Derived Neurons from Patients with 22q11.2 Deletion Syndrome and Schizophrenia. Transl. Psychiatry 9, 302. 10.1038/s41398-019-0643-y 31740674 PMC6861238

[B65] Li Q. Wang C. Tang H. Chen S. Ma J. (2018). Stroke and Coronary Artery Disease Are Associated with Parkinson's Disease. Can. J. Neurol. Sci. 45 (5), 559–565. 10.1017/cjn.2018.56 30001757

[B66] Liu P. Cheng Y. Xu H. Huang H. Tang S. Song F. (2021). TRESK Regulates Gm11874 to Induce Apoptosis of Spinal Cord Neurons via ATP5i Mediated Oxidative Stress and DNA Damage. Neurochem. Res. 46 (8), 1970–1980. 10.1007/s11064-021-03318-w 33973102

[B67] Lopez E. O. Ballard B. D. Jan A. (2021). Cardiovascular Disease. StatPearls [Internet].

[B68] Luo W. Wang Y. Zhang L. Ren P. Zhang C. Li Y. (2020). Critical Role of Cytosolic DNA and its Sensing Adaptor STING in Aortic Degeneration, Dissection, and Rupture. Circulation 141 (1), 42–66. 10.1161/CIRCULATIONAHA.119.041460 31887080 PMC6939474

[B69] Lusis A. J. (2000). Atherosclerosis. Nature. 407, 233–241. 10.1038/35025203 11001066 PMC2826222

[B70] Malekmohammad K. Bezsonov E. E. Rafieian-Kopaei M. (2021). Role of Lipid Accumulation and Inflammation in Atherosclerosis: Focus on Molecular and Cellular Mechanisms. Front. Cardiovasc. Med. 8, 707529. 10.3389/fcvm.2021.707529 34552965 PMC8450356

[B71] Manhas R. Rath P. C. (2020). Ribosome, Protein Synthesis, and Aging. Models, Mol. Mech. Biogerontology 2020, 67–87. 10.1007/978-981-32-9005-1_4

[B72] Margiotta A. (2021). Role of SNAREs in Neurodegenerative Diseases. Cells 10 (5), 991. 10.3390/cells10050991 33922505 PMC8146804

[B73] McCullagh K. J. Cooney R. O'Brien T. (2016). Endothelial Nitric Oxide Synthase Induces Heat Shock Protein HSPA6 (HSP70B') in Human Arterial Smooth Muscle Cells. Nitric Oxide 52, 41–48. 10.1016/j.niox.2015.11.002 26656590

[B74] Meng N. Chen K. Wang Y. Hou J. Chu W. Xie S. (2022). Dihydrohomoplantagin and Homoplantaginin, Major Flavonoid Glycosides from Salvia Plebeia R. Br. Inhibit oxLDL-Induced Endothelial Cell Injury and Restrict Atherosclerosis via Activating Nrf2 Anti-oxidation Signal Pathway. Molecules 27 (6), 1990. 10.3390/molecules27061990 35335352 PMC8951125

[B75] Merl-Pham J. Basak T. Knüppel L. Ramanujam D. Athanason M. Behr J. (2019). Quantitative Proteomic Profiling of Extracellular Matrix and Site-specific Collagen Post-translational Modifications in an *In Vitro* Model of Lung Fibrosis. Matrix Biol. Plus 1, 100005. 10.1016/j.mbplus.2019.04.002 33543004 PMC7852317

[B76] Mezzadra R. Sun C. Jae L. T. Gomez-Eerland R. de Vries E. Wu W. (2017). Identification of CMTM6 and CMTM4 as PD-L1 Protein Regulators. Nature 549 (7670), 106–110. 10.1038/nature23669 28813410 PMC6333292

[B77] Miller K. N. Victorelli S. G. Salmonowicz H. Dasgupta N. Liu T. Passos J. F. (2021). Cytoplasmic DNA: Sources, Sensing, and Role in Aging and Disease. Cell 184 (22), 5506–5526. 10.1016/j.cell.2021.09.034 34715021 PMC8627867

[B78] Moore K. J. Sheedy F. J. Fisher E. A. (2013). Macrophages in Atherosclerosis: a Dynamic Balance. Nat. Rev. Immunol. 13 (10), 709–721. 10.1038/nri3520 23995626 PMC4357520

[B79] Mota R. Parry T. L. Yates C. C. Qiang Z. Eaton S. C. Mwiza J. M. (2018). Increasing Cardiomyocyte Atrogin-1 Reduces Aging-Associated Fibrosis and Regulates Remodeling *In Vivo* . Am. J. Pathology 188 (7), 1676–1692. 10.1016/j.ajpath.2018.04.007 PMC6026801 29758183

[B80] Munger E. Hickey J. W. Dey A. K. Jafri M. S. Kinser J. M. Mehta N. N. (2021). Application of Machine Learning in Understanding Atherosclerosis: Emerging Insights. Apl. Bioeng. 5 (1), 011505. 10.1063/5.0028986 33644628 PMC7889295

[B81] Nakajima T. Yokota T. Shingu Y. Yamada A. Iba Y. Ujihira K. (2019). Impaired Mitochondrial Oxidative Phosphorylation Capacity in Epicardial Adipose Tissue Is Associated with Decreased Concentration of Adiponectin and Severity of Coronary Atherosclerosis. Sci. Rep. 9, 3535. 10.1038/s41598-019-40419-7 30837669 PMC6401184

[B82] Nickel W. Brügger B. Wieland F. T. (2002). Vesicular Transport: the Core Machinery of COPI Recruitment and Budding. J. Cell Sci. 115 (16), 3235–3240. 10.1242/jcs.115.16.3235 12140255

[B83] Nie Y. Cai S. Yuan R. Ding S. Zhang X. Chen L. (2020). Zfp422 Promotes Skeletal Muscle Differentiation by Regulating EphA7 to Induce Appropriate Myoblast Apoptosis. Cell Death Differ. 27 (5), 1644–1659. 10.1038/s41418-019-0448-9 31685980 PMC7206035

[B84] Niepiekło-Miniewska W. Żuk N. Dubis J. Kurpisz M. Senitzer D. Havrylyuk A. (2014). Two New Cases of KIR3DP1, KIR2DL4-Negative Genotypes, One of Which Is Also Lacking KIR3DL2. Arch. Immunol. Ther. Exp. 62 (5), 423–429. 10.1007/s00005-014-0299-5 PMC4164834 25033772

[B85] Nkoenig S. Cavus O. Williams J. Bernier M. Tonniges J. Sucharski H. (2022). New Mechanistic Insights to PLOD1-Mediated Human Vascular Disease. Transl. Res. 239, 1. 10.1016/j.trsl.2021.08.002 34400365 PMC8671190

[B86] Noonan E. J. Place R. F. Giardina C. Hightower L. E. (2007). Hsp70B′ Regulation and Function. Cell Stress Chaper 12, 393–402. 10.1379/csc-278e.1 PMC2134801 18229458

[B87] Ohki R. Nemoto J. Murasawa H. Oda E. Inazawa J. Tanaka N. (2000). Reprimo, a New Candidate Mediator of the P53-Mediated Cell Cycle Arrest at the G2 Phase. J. Biol. Chem. 275 (30), 22627–22630. 10.1074/jbc.C000235200 10930422

[B88] Osada K. Maeda Y. Yoshino T. Nojima D. Bowler C. Tanaka T. (2017). Enhanced NADPH Production in the Pentose Phosphate Pathway Accelerates Lipid Accumulation in the Oleaginous Diatom Fistulifera Solaris. Algal Res. 23, 126–134. 10.1016/j.algal.2017.01.015

[B89] Pahwa R. Jialal I. (2021). Atherosclerosis. StatPearls [Internet].

[B90] Patel R. P. Moellering D. Murphy-Ullrich J. Jo H. Beckman J. S. Darley-Usmar V. M. (2000). Cell Signaling by Reactive Nitrogen and Oxygen Species in Atherosclerosis. Free Radic. Biol. Med. 28 (12), 1780–1794. 10.1016/s0891-5849(00)00235-5 10946220

[B91] Privratsky J. R. Newman P. J. (2014). PECAM-1: Regulator of Endothelial Junctional Integrity. Cell Tissue Res. 355 (3), 607–619. 10.1007/s00441-013-1779-3 24435645 PMC3975704

[B92] Pugh J. Nemat-Gorgani N. Djaoud Z. Guethlein L. A. Norman P. J. Parham P. (2019). *In Vitro* education of Human Natural Killer Cells by KIR3DL1. Life Sci. Alliance 2 (6), e201900434. 10.26508/lsa.201900434 31723004 PMC6856763

[B93] Pushparaj P. N. Tay H. K. Wang C.-C. Hong W. Melendez A. J. (2009). VAMP8 Is Essential in Anaphylatoxin-Induced Degranulation, TNF-α Secretion, Peritonitis, and Systemic Inflammation. J. Immunol. 183 (2), 1413–1418. 10.4049/jimmunol.0804061 19564343

[B94] Rana M. Kumar A. Tiwari R. L. Singh V. Chandra T. Dikshit M. (2016). IRAK Regulates Macrophage Foam Cell Formation by Modulating Genes Involved in Cholesterol Uptake and Efflux. Bioessays 38 (7), 591–604. 10.1002/bies.201600085 27270491

[B95] Récher C. (2021). Clinical Implications of Inflammation in Acute Myeloid Leukemia. Front. Oncol. 11, 623952. 10.3389/fonc.2021.623952 33692956 PMC7937902

[B96] Röning T. Magga J. Anna L. Kerkelä R. Koivunen P. Serpi R. (2022). Activation of the Hypoxia Response Pathway Protects against Age-Induced Cardiac Hypertrophy. J. Mol. Cell Cardiol. 164, 148–155. 10.1016/j.yjmcc.2021.12.003 34919895

[B97] Ross K. A. (2014). Coherent Somatic Mutation in Autoimmune Disease. PLoS ONE 9 (7), e101093. 10.1371/journal.pone.0101093 24988487 PMC4079513

[B98] Sadighi Akha A. A. (2018). Aging and the Immune System: An Overview. J. Immunol. Methods 463, 21–26. 10.1016/j.jim.2018.08.005 30114401

[B99] Sainlos M. Belmont P. Vigneron J.-P. Lehn P. Lehn J.-M. (2003). Aminoglycoside-Derived Cationic Lipids for Gene Transfection: Synthesis of Kanamycin A Derivatives. Eur. J. Org. Chem. 2003 (15), 2764–2774. 10.1002/ejoc.200300164

[B100] Samanta D. Huang T. Y.-T. Shah R. Yang Y. Pan F. Semenza G. L. (2020). BIRC2 Expression Impairs Anti-cancer Immunity and Immunotherapy Efficacy. Cell Rep. 32 (8), 108073. 10.1016/j.celrep.2020.108073 32846130

[B101] Sánchez-Cabo F. Rossello X. Fuster V. Benito F. Manzano J. P. Silla J. C. (2020). Machine Learning Improves Cardiovascular Risk Definition for Young, Asymptomatic Individuals. J. Am. Coll. Cardiol. 76 (14), 1674–1685. 10.1016/j.jacc.2020.08.017 33004133

[B102] Santoro M. M. Samuel T. Mitchell T. Reed J. C. Stainier D. Y. R. (2007). Birc2 (cIap1) Regulates Endothelial Cell Integrity and Blood Vessel Homeostasis. Nat. Genet. 39, 1397–1402. 10.1038/ng.2007.8 17934460

[B103] Sayed N. Huang Y. Nguyen K. Krejciova-Rajaniemi Z. Grawe A. P. Gao T. (2021). An Inflammatory Aging Clock (iAge) Based on Deep Learning Tracks Multimorbidity, Immunosenescence, Frailty and Cardiovascular Aging. Nat. Aging 1, 598–615. 10.1038/s43587-021-00082-y 34888528 PMC8654267

[B104] Schisler J. C. Grevengoed T. J. Pascual F. Cooper D. E. Ellis J. M. Paul D. S. (2015). Cardiac Energy Dependence on Glucose Increases Metabolites Related to Glutathione and Activates Metabolic Genes Controlled by Mechanistic Target of Rapamycin. Jaha 4 (2), e001136. 10.1161/JAHA.114.001136 25713290 PMC4345858

[B105] Shadrina A. S. Shashkova T. I. Torgasheva A. A. Sharapov S. Z. Klarić L. Pakhomov E. D. (2020). Prioritization of Causal Genes for Coronary Artery Disease Based on Cumulative Evidence from Experimental and In Silico Studies. Sci. Rep. 10, 10486. 10.1038/s41598-020-67001-w 32591598 PMC7320185

[B106] Shi Q. Chen L. (2017). Cereblon: A Protein Crucial to the Multiple Functions of Immunomodulatory Drugs as Well as Cell Metabolism and Disease Generation. J. Immunol. Res. 2017, 1–8. 10.1155/2017/9130608 PMC5574216 28894755

[B107] Shimizu H. Taniguchi T. Ishikawa Y. Yokoyama M. (1997). Effects of Nitric Oxide on Cholesterol Metabolism in Macrophages. Atherosclerosis 129 (2), 193–198. 10.1016/s0021-9150(96)06032-7 9105561

[B108] Shirvani S. M. Mookanamparambil L. Ramoni M. F. Chin M. T. (2007). Transcription Factor CHF1/Hey2 Regulates the Global Transcriptional Response to Platelet-Derived Growth Factor in Vascular Smooth Muscle Cells. Physiol. Genomics 30, 61. 10.1152/physiolgenomics.00277.2006 17327490

[B109] Singer J. W. Fleischman A. Al-Fayoumi S. Mascarenhas J. O. Yu Q. Agarwal A. (2018). Inhibition of Interleukin-1 Receptor-Associated Kinase 1 (IRAK1) as a Therapeutic Strategy. Oncotarget 9 (70), 33416–33439. 10.18632/oncotarget.26058 30279971 PMC6161786

[B110] So J. S. (2018). Roles of Endoplasmic Reticulum Stress in Immune Responses. Mol. Cells 41 (8), 705–716. 10.14348/molcells.2018.0241 30078231 PMC6125421

[B111] Stadtman E. R. Van Remmen H. Richardson A. Wehr N. B. Levine R. L. (2005). Methionine Oxidation and Aging. Biochimica Biophysica Acta (BBA) - Proteins Proteomics 1703 (2), 135–140. 10.1016/j.bbapap.2004.08.010 15680221

[B112] Stein K. C. Morales-Polanco F. van der Lienden J. Rainbolt T. K. Frydman J. (2022). Ageing Exacerbates Ribosome Pausing to Disrupt Cotranslational Proteostasis. Nature 601 (7894), 637–642. 10.1038/s41586-021-04295-4 35046576 PMC8918044

[B113] Stewart J. Manmathan G. Wilkinson P. (2017). Primary Prevention of Cardiovascular Disease: A Review of Contemporary Guidance and Literature. JRSM Cardiovasc. Dis. 6, 204800401668721. 10.1177/2048004016687211 PMC5331469 28286646

[B114] Swirski F. K. Nahrendorf M. (2014). Leukocyte Behavior in Atherosclerosis, Myocardial Infarction, and Heart Failure. Science 339, 161. 10.1126/science.1230719 PMC3891792 23307733

[B115] Takai T. (2002). Roles of Fc Receptors in Autoimmunity. Nat. Rev. Immunol. 2, 580–592. 10.1038/nri856 12154377

[B116] Toba H. Cannon P. L. Yabluchanskiy A. Iyer R. P. D’Armiento J. Lindsey M. L. (2017). Transgenic Overexpression of Macrophage Matrix Metalloproteinase-9 Exacerbates Age-Related Cardiac Hypertrophy, Vessel Rarefaction, Inflammation, and Fibrosis. Am. J. Physiology-Heart Circulatory Physiology 312, H375–H383. 10.1152/ajpheart.00633.2016 PMC5402013 28011588

[B117] Tokarev A. A. Alfonso A. Segev N. (2009). “Overview of Intracellular Compartments and Trafficking Pathways,” in Trafficking inside Cells. Molecular Biology Intelligence Unit (New York, NY: Springer). 10.1007/978-0-387-93877-6_1

[B118] Turi Z. Lacey M. Mistrik M. Moudry P. (2019). Impaired Ribosome Biogenesis: Mechanisms and Relevance to Cancer and Aging. Aging 11, 2512–2540. 10.18632/aging.101922 31026227 PMC6520011

[B119] Tyrrell D. J. Goldstein D. R. (2020). Ageing and Atherosclerosis: Vascular Intrinsic and Extrinsic Factors and Potential Role of IL-6. Nat. Rev. Cardiol. 18, 58–68. 10.1038/s41569-020-0431-7 32918047 PMC7484613

[B120] Van-Assche T. Huygelen V. Crabtree M. J. Antoniades C. (2011). Gene Therapy Targeting Inflammation in Atherosclerosis. Curr. Pharm. Des. 17 (37), 4210–4223. 10.2174/138161211798764799 22204379

[B121] Wågsäter D. Paloschi V. Hanemaaijer R. Hultenby K. Bank R. A. Franco‐Cereceda A. (2013). Impaired Collagen Biosynthesis and Cross‐linking in Aorta of Patients with Bicuspid Aortic Valve. Jaha 2 (1), e000034. 10.1161/JAHA.112.000034 23525417 PMC3603268

[B122] Wang J. C. Bennett M. (2012). Aging and Atherosclerosis. Circ. Res. 111 (2), 245–259. 10.1161/CIRCRESAHA.111.261388 22773427

[B123] Wang L. Hou J. Wang J. Zhu Z. Zhang W. Zhang X. (2020). Regulatory Roles of HSPA6 in Actinidia Chinensis Planch. Root Extract (acRoots)‐inhibited Lung Cancer Proliferation. Clin. Transl. Med. 10, 2. 10.1002/ctm2.46 PMC7403824 32508044

[B124] Wang X. Chen T. Deng Z. Gao W. Liang T. Qiu X. (2021). Melatonin Promotes Bone Marrow Mesenchymal Stem Cell Osteogenic Differentiation and Prevents Osteoporosis Development through Modulating Circ_0003865 that Sponges miR-3653-3p. Stem Cell Res. Ther. 12, 150. 10.1186/s13287-021-02224-w 33632317 PMC7908669

[B125] Wang Y. Huang T. Li Y. Sha X. (2020). The Self-Organization Model Reveals Systematic Characteristics of Aging. Theor. Biol. Med. Model. 17, 4. 10.1186/s12976-020-00120-z 32197622 PMC7082995

[B126] Wei T. Liu J. Zhang D. Wang X. Li G. Ma R. (2021). The Relationship between Nutrition and Atherosclerosis. Front. Bioeng. Biotechnol. 9, 635504. 10.3389/fbioe.2021.635504 33959594 PMC8094392

[B127] Wu W. Yu N. Li F. Gao P. Lin S. Zhu Y. (2021). RPL35 Promotes Neuroblastoma Progression via the Enhanced Aerobic Glycolysis. Am. J. Cancer Res. 11 (11), 5701–5714. 34873488 PMC8640819

[B128] Xia S. Zhang X. Zheng S. Khanabdali R. Kalionis B. Wu J. (2016). An Update on Inflamm-Aging: Mechanisms, Prevention, and Treatment. J. Immunol. Res. 2016, 1–12. 10.1155/2016/8426874 PMC4963991 27493973

[B129] Xu M. Knox A. J. Michaelis K. A. Kiseljak-Vassiliades K. Kleinschmidt-De B. K. Lillehei K. O. (2012). Reprimo (RPRM) Is a Novel Tumor Suppressor in Pituitary Tumors and Regulates Survival, Proliferation, and Tumorigenicity. Endocrinology 153 (7), 2963–2973. 10.1210/en.2011-2021 22562171 PMC4714648

[B130] Yang J. Boerm M. McCarty M. Bucana C. Fidler I. J. Z J Yang Y. (2000). Mekk3 Is Essential for Early Embryonic Cardiovascular Development. Nat. Genet. 24 (3), 309–313. 10.1038/73550 10700190

[B131] Yazdani A. Yazdani A. Samiei A. Boerwinkle E. (2016). Generating a Robust Statistical Causal Structure over 13 Cardiovascular Disease Risk Factors Using Genomics Data. J. Biomed. Inf. 60 (C), 114–119. 10.1016/j.jbi.2016.01.012 PMC4886234 26827624

[B132] Ye G.-C. Liu Y.-F. Huang L. Zhang C.-Y. Sheng Y.-L. Wu B. (2021). Key microRNAs and Hub Genes Associated with Poor Prognosis in Lung Adenocarcinoma. Aging 13 (3), 3742–3762. 10.18632/aging.202337 33461176 PMC7906143

[B133] Yin J. Ibrahim S. Petersen F. Yu X. (2021). Autoimmunomic Signatures of Aging and Age-Related Neurodegenerative Diseases Are Associated with Brain Function and Ribosomal Proteins. Front. Aging Neurosci. 13, 679688. 10.3389/fnagi.2021.679688 34122052 PMC8192960

[B134] Yu J. Mursu E. Typpö M. Laaksonen S. Voipio H.-M. Pesonen P. (2019). MMP-3 and MMP-8 in Rat Mandibular Condylar Cartilage Associated with Dietary Loading, Estrogen Level, and Aging. Archives Oral Biol. 97, 238–244. 10.1016/j.archoralbio.2018.10.037 30412863

[B135] Yuan B. Xu Y. Zheng S. (2021). PLOD1 Acts as a Tumor Promoter in Glioma via Activation of the HSF1 Signaling Pathway. Mol. Cell. Biochem. 477, 549. 10.1007/s11010-021-04289-w 34845571

[B136] Zhang R. (2013). MNADK, a Novel Liver-Enriched Mitochondrion-Localized NAD Kinase. Biol. Open 2 (4), 432–438. 10.1242/bio.20134259 23616928 PMC3625872

[B137] Zhang Y. Wu Y. Su X. (2021). PLOD1 Promotes Cell Growth and Aerobic Glycolysis by Regulating the SOX9/PI3K/Akt/mTOR Signaling Pathway in Gastric Cancer. Front. Bioscience-Landmark 26, 322. 10.52586/4946 34455762

[B138] Zhavoronkov A. Mamoshina P. Vanhaelen Q. Scheibye-Knudsen M. Moskalev A. Aliper A. (2019). Artificial Intelligence for Aging and Longevity Research: Recent Advances and Perspectives. Ageing Res. Rev. 49, 49–66. 10.1016/j.arr.2018.11.003 30472217

[B139] Zheng L. Xu H. Di Y. Chen L. Liu J. Kang L. (2021). ELK4 Promotes the Development of Gastric Cancer by Inducing M2 Polarization of Macrophages through Regulation of the KDM5A-PJA2-KSR1 axis. J. Transl. Med. 19, 342. 10.1186/s12967-021-02915-1 34372882 PMC8353876

[B140] Zheng Y. Wang C. Song A. Jiang F. Zhou J. Li G. (2020). CMTM6 Promotes Cell Proliferation and Invasion in Oral Squamous Cell Carcinoma by Interacting with NRP1. Am. J. Cancer Res. 10 (6), 1691–1709. 32642284 PMC7339282

[B141] Zheng Y. Liu H. Kong Y. (2017). miR-188 Promotes Senescence of Lineage-Negative Bone Marrow Cells by Targeting MAP3K3 Expression. FEBS Lett. 591 (15), 2290–2298. 10.1002/1873-3468.12720 28640956

