# Supplementary material for: The Inflamm-Aging Model Identifies Key Risk Factors in Atherosclerosis
Source: Front Genet. 2022 May 30;13:865827. doi: 10.3389/fgene.2022.865827 (PMC9191626; doi:10.3389/fgene.2022.865827)
Supplement: Supplementary file 1 [file DataSheet1.ZIP › Supplemental files/TableS8-S14.docx]

**Table S8** The accuracy of the training dataset and test dataset for the predictor used SVM

|  | The accurcy for training dataset | The accurcy for test dataset |
| --- | --- | --- |
| The traditional aging predictor | 0.9964 | 0.5611 |
| The aging predictor by our previous study | 0.9964 | 0.7066 |
| The traditional disease predictor | 0.7932 | 0.5518 |
| The disease predictor by our previous study | 0.9471 | 0.5775 |
| The improved inflamm-aging predictor | 0.9071 | 0.5882 |

**Table S9** The accuracy of the training dataset and test dataset for the predictor used ensemble

|  | The accurcy for training dataset | The accurcy for test dataset |
| --- | --- | --- |
| The traditional aging predictor | 0.9964 | 0.6525 |
| The aging predictor by our previous study | 0.5000 | 0.6593 |
| The traditional disease predictor | 0.9057 | 0.5973 |
| The disease predictor by our previous study | 1 | 0.6012 |
| The improved inflamm-aging predictor | 1 | 0.6181 |

**Table S10** The accuracy of the training dataset and test dataset for the predictor used naive bayes

|  | The accurcy for training dataset | The accurcy for test dataset |
| --- | --- | --- |
| The traditional aging predictor | 0.7833 | 0.5850 |
| The aging predictor by our previous study | 0.8131 | 0.6514 |
| The traditional disease predictor | 0.7432 | 0.6085 |
| The disease predictor by our previous study | 0.7421 | 0.5988 |
| The improved inflamm-aging predictor | 0.7389 | 0.5988 |

**TableS11** The aging marker related to the disease markers

| Aging marker | Times |
| --- | --- |
| RPRM | 39 |
| ICAM2 | 35 |
| CRBN | 35 |
| PDC | 34 |
| CCNB1IP1 | 34 |
| ZNF654 | 34 |
| HEY2 | 33 |
| BIRC2 | 29 |
| CKAP4 | 27 |
| BCR | 27 |

**TableS12** The inflamm-aging marker related to the aging markers

| Inflamm-aging marker | Times |
| --- | --- |
| PECAM1 | 53 |
| CEBPA | 49 |
| PARP16 | 45 |
| POM121 | 43 |
| ELP3 | 41 |
| UBE2G2 | 40 |
| PLOD1 | 37 |
| KIR2DS1 | 36 |
| VPS16 | 34 |
| SPATA2 | 30 |

**TableS13** The aging marker related to the disease markers in sensitivity analysis

| Aging marker | Times |
| --- | --- |
| HEY2 | 16 |
| CRBN | 14 |
| ICAM2 | 12 |
| ZNF654 | 12 |
| BIRC2 | 11 |
| DLG3 | 11 |
| PDC | 11 |
| RPRM | 11 |
| CKAP4 | 10 |
| SLC4A1 | 10 |

**TableS14** The inflamm-aging marker related to the aging markers in sensitivity analysis

| Inflamm-aging marker | Times |
| --- | --- |
| PECAM1 | 23 |
| CEBPA | 18 |
| POM121 | 17 |
| PARP16 | 13 |
| CPNE1 | 12 |
| ELP3 | 12 |
| SDHC | 11 |
| RBBP5 | 11 |
| DAZL | 11 |
| PLOD1 | 10 |
