# Supplementary figures and images for: The Inflamm-Aging Model Identifies Key Risk Factors in Atherosclerosis
Source: Front Genet. 2022 May 30;13:865827. doi: 10.3389/fgene.2022.865827 (PMC9191626; doi:10.3389/fgene.2022.865827)

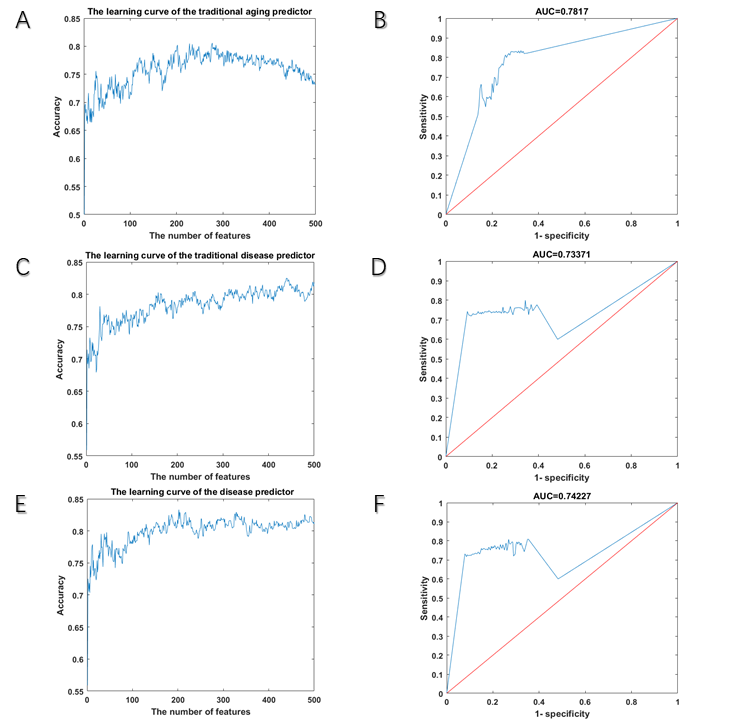

Supplement: Supplementary file 1 [file DataSheet1.ZIP › Supplemental files/Figure S1.tif]

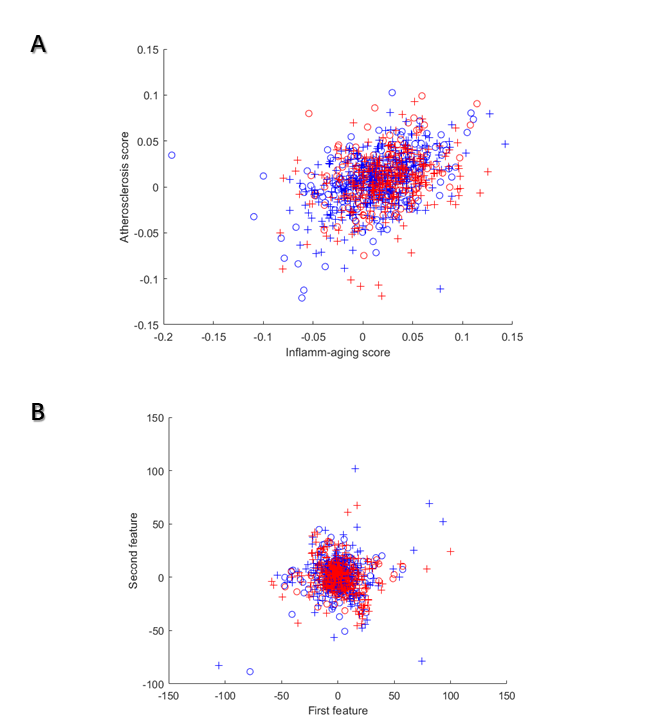

Supplement: Supplementary file 1 [file DataSheet1.ZIP › Supplemental files/Figure S2.tif]

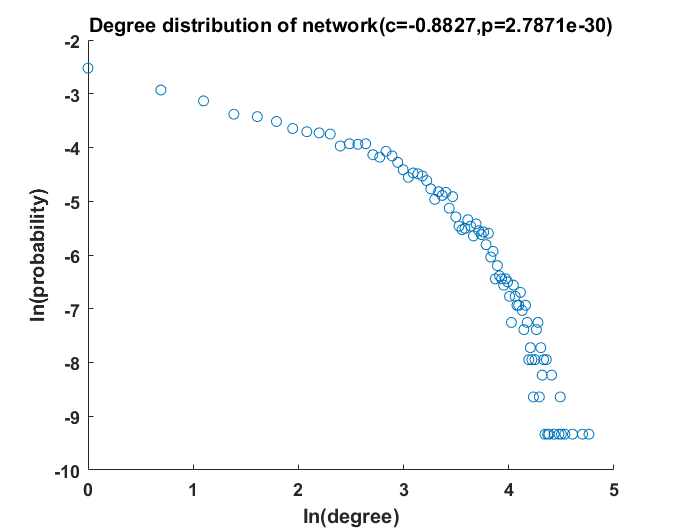

Supplement: Supplementary file 1 [file DataSheet1.ZIP › Supplemental files/Figure S3.tif]

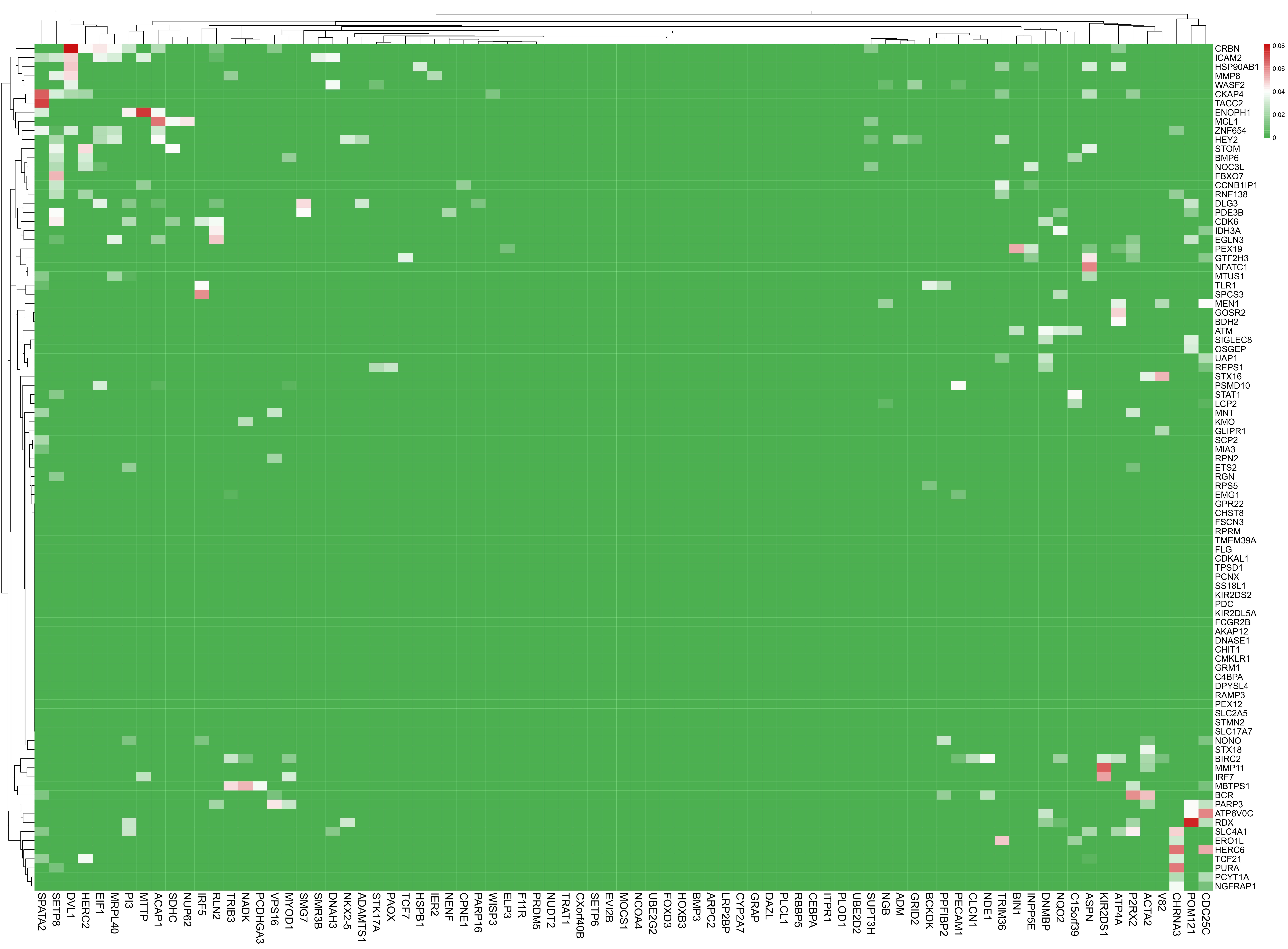

Supplement: Supplementary file 1 [file DataSheet1.ZIP › Supplemental files/Figure S4.tif]

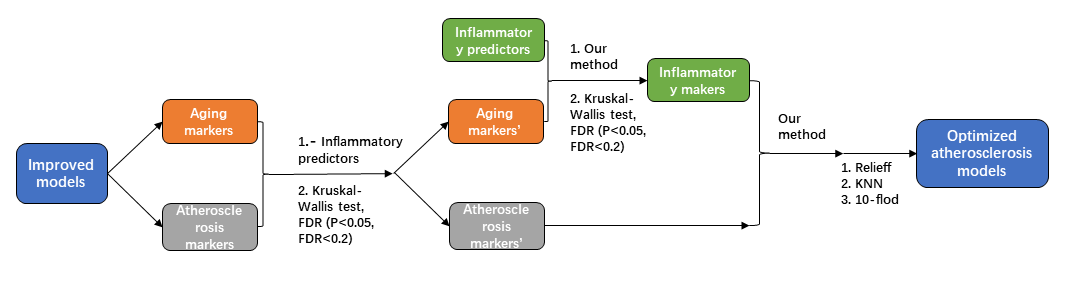

Supplement: Supplementary file 1 [file DataSheet1.ZIP › Supplemental files/Figure S5.tif]
